# PRDM15 is a key regulator of metabolism critical to sustain B-cell lymphomagenesis

**DOI:** 10.1038/s41467-020-17064-0

**Published:** 2020-07-14

**Authors:** Slim Mzoughi, Jia Yi Fong, David Papadopoli, Cheryl M. Koh, Laura Hulea, Paolo Pigini, Federico Di Tullio, Giuseppe Andreacchio, Michal Marek Hoppe, Heike Wollmann, Diana Low, Matias J. Caldez, Yanfen Peng, Denis Torre, Julia N. Zhao, Oro Uchenunu, Gabriele Varano, Corina-Mihaela Motofeanu, Manikandan Lakshmanan, Shun Xie Teo, Cheng Mun Wun, Giovanni Perini, Soo Yong Tan, Chee Bing Ong, Muthafar Al-Haddawi, Ravisankar Rajarethinam, Susan Swee-Shan Hue, Soon Thye Lim, Choon Kiat Ong, Dachuan Huang, Siok-Bian Ng, Emily Bernstein, Dan Hasson, Keng Boon Wee, Philipp Kaldis, Anand Jeyasekharan, David Dominguez-sola, Ivan Topisirovic, Ernesto Guccione

**Affiliations:** 1 Institute of Molecular and Cell Biology (IMCB), Agency for Science, Technology and Research (A*STAR), Singapore, Singapore; 2 Department of Biochemistry, Yong Loo Lin School of Medicine, National University of Singapore, Singapore, Singapore; 3 Department of Oncological Sciences, Tisch Cancer Institute, Icahn School of Medicine at Mount Sinai, New York, NY USA; 4 NUS Graduate School for Integrative Sciences and Engineering, National University of Singapore, Singapore, Singapore; 5 Lady Davis Institute, SMBD JGH, McGill University, Gerald Bronfman Department of Oncology, McGill University, Montreal, QC H3T 1E2 Canada; 6 Maisonneuve-Rosemont Hospital Research Centre, 5415 Assumption Blvd, Montreal, QC H1T 2M4 Canada; 7 Département de Médecine, Université de Montréal, CP 6128, Succursale Centre-Ville, Montréal, QC H3C 3J7 Canada; 8 Department of Pharmacy and Biotechnology, University of Bologna, Via F. Selmi 3, 40126 Bologna, Italy; 9 Cancer Science Institute (CSI), National University of Singapore, Singapore, Singapore; 10 Immunology Institute and Graduate School of Biomedical Sciences, Icahn School of Medicine at Mount Sinai, New York, NY USA; 11 Advanced Molecular Pathology Laboratory, IMCB, Singapore, Singapore; 12 Department of Pathology, Yong Loo Lin School of Medicine, National University of Singapore, Singapore, Singapore; 13 National University Hospital (NUH), Singapore, Singapore; 14 Division of Medical Oncology, National Cancer Centre Singapore, Singapore, Singapore; 15 Duke-NUS Graduate Medical School, Singapore, Singapore; 16 Division of Cellular and Molecular Research, National Cancer Centre Singapore, Singapore, Singapore; 17 Genome Institute of Singapore (GIS), Agency for Science, Technology and Research (A*STAR), Singapore, Singapore; 18 Lady Davis Institute, SMBD JGH, McGill University, Departments of Experimental Medicine and Biochemistry, McGill University, Montreal, QC H3T 1E2 Canada; 19 Mount Sinai Center for Therapeutics Discovery, Department of Oncological and Pharmacological Sciences, Icahn School of Medicine at Mount Sinai, New York, NY USA; 20 Present Address: Immunology Frontiers Research Center, Osaka University, 3-1 Yamada-oka, Suita, 565-0871 Japan

**Keywords:** B-cell lymphoma, Transcriptomics

## Abstract

PRDM (PRDI-BF1 and RIZ homology domain containing) family members are sequence-specific transcriptional regulators involved in cell identity and fate determination, often dysregulated in cancer. The *PRDM15* gene is of particular interest, given its low expression in adult tissues and its overexpression in B-cell lymphomas. Despite its well characterized role in stem cell biology and during early development, the role of PRDM15 in cancer remains obscure. Herein, we demonstrate that while PRDM15 is largely dispensable for mouse adult somatic cell homeostasis in vivo, it plays a critical role in B-cell lymphomagenesis. Mechanistically, PRDM15 regulates a transcriptional program that sustains the activity of the PI3K/AKT/mTOR pathway and glycolysis in B-cell lymphomas. Abrogation of PRDM15 induces a metabolic crisis and selective death of lymphoma cells. Collectively, our data demonstrate that PRDM15 fuels the metabolic requirement of B-cell lymphomas and validate it as an attractive and previously unrecognized target in oncology.

## Abstract (web summary)

The transcriptional regulator PRDM15 is expressed at low levels in normal tissues but overexpressed in B-cell lymphomas. Here, the authors show that PRDM15 depletion does not affect adult somatic cell homeostasis but leads to a metabolic crisis which impairs B-cell lymphomagenesis.

## Introduction

Non-Hodgkin’s lymphomas (NHLs) are the most prevalent form of adult hematological cancers, with B-cell lymphomas accounting for the majority (>85%) of all cases^1^. Diffuse large B-cell lymphoma (DLBCL) is a heterogeneous group of aggressive B-cell neoplasms, of which only 60% of cases are curable with standard R-CHOP therapy (rituximab, cyclophosphamide, doxorubicin, vincristine, prednisone)^2^. Around 30% of DLBCLs show either upregulation, stabilization or translocation of *MYC*, with or without a second or third translocation involving *BCL2* and *BCL6*, respectively^3–7^. This is associated to very aggressive clinical presentation and dismal outcomes.

Given the diversity of NHLs, there is an unmet need to identify proteins that can be targeted for therapeutic intervention across a wide range of aggressive lymphomas. These targets should be tractable, and their depletion should have minimal adverse effects on normal hematopoiesis.

The PRDM family of transcription factors is extremely attractive from a therapeutic perspective due to the presence of C_2_H_2_ Zinc Fingers at the C-terminus (conferring sequence specificity)^8,9^ and a PR domain at the N-terminus (conferring enzymatic activity and tractability)^10^. The PR domain is functionally and structurally related to the SET domain, which is the catalytic domain of protein lysine methyltransferases^11^. Indeed, some, but not all PR domains have been shown to directly methylate lysine residues (summarized in ref. ^12^).

Evidence from multiple studies suggest that PRDM family members, while sharing a common structure, bind to unique motifs on chromatin, and exert distinct functions^12^. Notably, many PRDMs have been linked to cancer initiation and/or maintenance. Examples include: (i) PRDM1/BLIMP1, a well-characterized tumor suppressor in DLBCL and other hematological tumors^13,14^. PRDM1 expression is often silenced in activated-B-cell-like (ABC)-DLBCL by multiple genetic and epigenetic mechanisms^15,16^, which prevents the terminal differentiation and increases the proliferative capacity of B cells^14^. Conversely, PRDM1 overexpression induced a G1 cell cycle arrest in DLBCL cells; (ii) The genomic locus containing *PRDM2* (Chr.1p36) is frequently deleted or rearranged in multiple cancer types^17–19^. Interestingly, *Prdm2*-null mice develop diffuse large B-cell lymphomas (DLBCL)^20^, while its overexpression induces p53-dependent apoptosis^21^; (iii) The PRDM14 locus (8q13) is amplified in nearly 25% of human lymphoid neoplasms^22,23^; (iv) Last, to date there is no evidence that PRDM15 has any functional role in tumorigenesis, despite its documented overexpression in follicular lymphoma (FL)^24^.

Herein, we demonstrate that PRDM15 is upregulated not only in FL, but in the majority of B-cell lymphomas. Antisense oligonucleotide (AON)-mediated depletion of PRDM15 impairs tumor growth in a lymphoma PDX model. Furthermore, genetic deletion of *Prdm15* in the EµMyc mouse model abolishes B-cell lymphomagenesis. Conversely, PRDM15 is largely dispensable for mouse adult somatic cell homeostasis in vivo, thus suggesting a wide therapeutic window. Mechanistically, PRDM15 regulates the transcription of key upstream regulators of the PI3K/AKT/mTOR pathway (*Igf1R*, *InsR*) and glycolysis (i.e.*, Eno3, Gapdh, Pkm, Pfkp,* and *Hk3*). Its depletion, both in vitro and in vivo, leads to a metabolic crisis and cell death in lymphoma, but not in normal cells.

Collectively, these data demonstrate that PRDM15 acts as a master regulator controlling the transcription of key metabolic enzymes and regulators, to fuel B-cell lymphomagenesis, and validate it as a promising and hitherto unrecognized target for the treatment of B-cell lymphomas.

## Results

### PRDM15 overexpression is critical for maintenance of human lymphomas

**Fig. 1:**
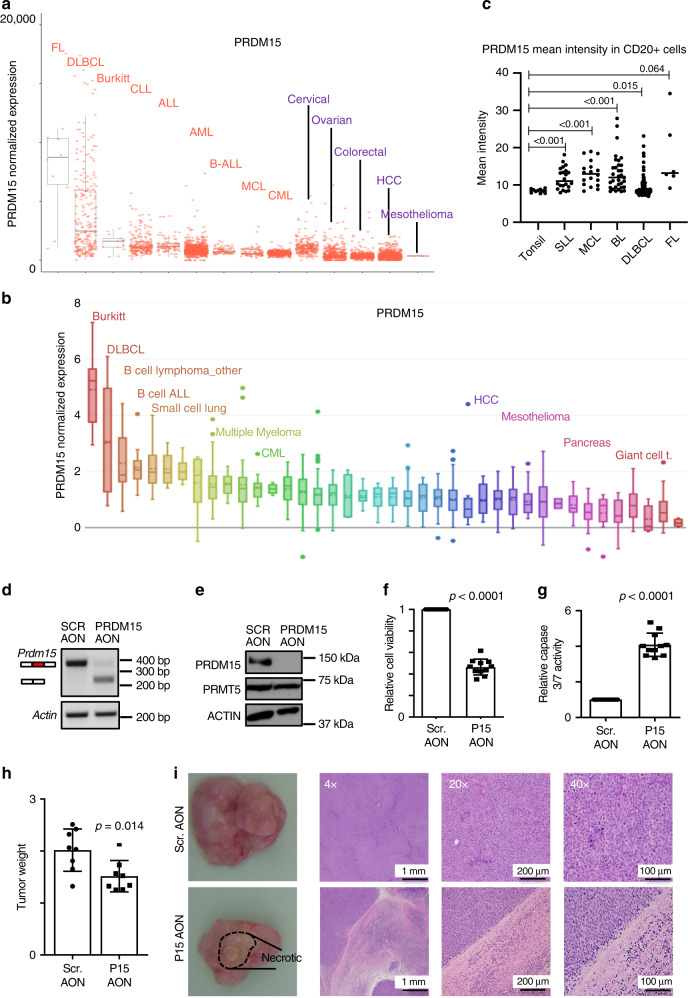
PRDM15 is overexpressed in human lymphomas and sustains tumor growth. Expression of PRDM15 across **a** Human specimens and cell lines available from GEO/SRA datasets and **b** multiple cell lines (source: Broad CCLE-https://portals.broadinstitute.org/ccle). In panels **a** and **b**, the lower and upper portions of the box plots outline the 25th (Q1) and 75th (Q3) percentile values. Centre line is the median (50th percentile (Q2). Crossbar lines at each whisker boundary show the minima (Q1−1.5*IQR) and maxima (Q3 + 1.5*IQR). **c** PRDM15 expression in normal tonsil and lymphoma tissue assessed by quantitative IHC. Each dot is the mean value of all cells in a single case; lines represent mean with 95% CI, error bars, s.d.; *n* = 6 (FL); *n* = 22 (SLL), *n* = 18 (MCL), *n* = 33 (Burkitt), *n* = 142 (DLBCL). Statistical significance was assessed using unpaired *t* test with Welch’s correction, two-tailed *p* value. **d** Semi-quantitative PCR to assess skipping of exon15 by the PRDM15 Antisense Oligo Nucleotide. **e** Validation of PRDM15 reduction by western blotting. PRMT5 and ACTIN are negative controls for AON specificity. **f** Relative viability and **g** relative Caspase 3/7 activity in patient-derived DLBCL cells 3 days following electroporation with the indicated AONs (*n* = 11 independent cultures). **h** Cohorts of a PDX model of relapsed DLBCL were established (*n* = 8). When tumors reached 150–250 mm^3^ of average volume, they were treated with either scrambled or PRDM15 AONs intratumorally every 2 days. Tumor size was assessed at day 21. **f–h** Data represent the mean (±SD). Student’s *t* test (two-sided) was used. **i** Representative gross (left panels) and H&E images (right panels) of the tumors treated with Scrambled AON and PRDM15 AON (*n* = 8). In SCR-AON tumors, the diffuse sheet of large round neoplastic cells contained round to oval vesicular nuclei with prominent nucleoli and numerous mitotic index. PRDM15-AON-treated tumor xenografts had a large (~50%) area of necrosis infiltrated by mixed inflammatory cells. CLL chronic lymphocytic leukemia, MCL mantle cell lymphoma, BL Burkitt’s lymphoma, DLBCL diffuse large B-cell lymphoma, FL follicular lymphoma.

Notwithstanding that it was recently reported that *PRDM15* is highly expressed in immune cells and overexpressed in FL^24^, its function in cancer remains largely undocumented. Intrigued by this observation, we first assessed the expression of *PRDM15* mRNA across cancer cell lines and patient samples. *PRDM15* is highly expressed, but rarely mutated, in FL^24^, DLBCL and Burkitt’s lymphomas (BL) (Fig. 1a and Supplementary Fig. 1A) and in B-cell-derived lymphoma cell lines (i.e. Burkitt’s, DLBCL, B-ALL, etc.) (Fig. 1b). Staining of a B-cell lymphoma-tissue microarray (TMA) confirmed elevated levels of PRDM15, and nuclear localization, in FL, DLBCL, BL, small lymphocytic-lymphomas (SLL) and mantel cell-lymphomas (MCL), compared to normal tonsil controls (Fig. 1c and Supplementary Fig. 1B, C).

These findings prompted us to investigate a potential functional role for PRDM15 in lymphomagenesis. We designed AONs to induce the skipping of exon 15 of human *PRDM15* mRNA, which is predicted to induce non-sense-mediated decay (NMD). The most efficient *PRDM15*-specific AON (IC_50_ = 25 nM) (Supplementary Fig. 2A and B) induced the skipping of exon 15 (Fig. 1d),subsequently reducing PRDM15 protein levels (Fig. 1e) in a primary patient-derived relapsed DLBCL. In contrast, the levels of the housekeeping protein, β-actin, or the arginine methyltransferase PRMT5^25^ were not affected, thus indicating AON specificity (Fig. 1e). Strikingly, depletion of PRDM15 induced apoptosis in primary DLBCL cells (Fig. 1f), as evidenced by the increase in caspase 3/7 activity (Fig. 1g). We confirmed these results in vivo, whereby intratumorally injected *PRDM15* AON reduced tumor weight (Fig. 1h), at least in part due to necrosis (Fig. 1i and Supplementary Fig. 2C). Similar results were observed in various established B-cell lymphoma lines, including P493-6 (BL-like), MC116 (undifferentiated lymphoma), OCILY3, Karpas231, PR1, and HT (DLBCL) (Supplementary Fig. 2D–F). Collectively, these findings suggest that PRDM15 may play a major role in B-cell lymphoma maintenance.

### PRDM15 is dispensable for normal adult murine homeostasis

**Fig. 2:**
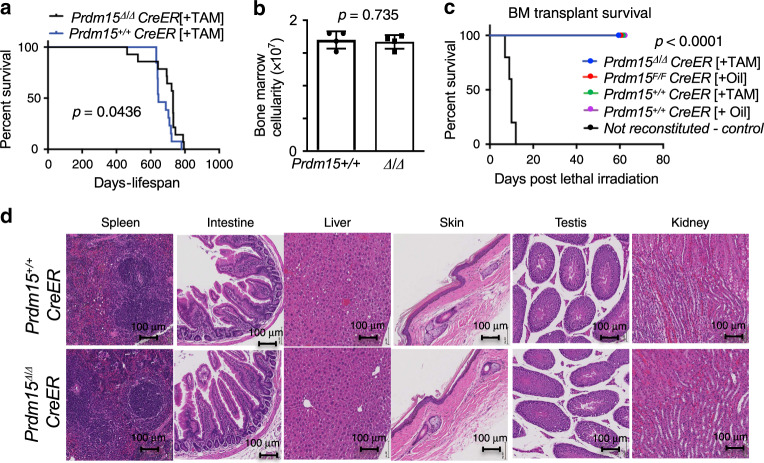
PRDM15 is largely dispensable for normal adult murine homeostasis. **a** Survival curve of *PRDM15*^*+/+*^*Rosa26;CreER* (*n* = 13) or *PRDM15*^*F/F*^*;Rosa26;CreER* (*n* = 14) mice that were injected with Tamoxifen (TAM) at 8 weeks of age (*Prdm15*^∆/∆^*)*. **b** Bone marrow cellularity and white blood (WBC) cell counts of PRDM15+/+ and PRDM15∆/∆ mice 2 months after tamoxifen injection (*n* = 4). Center values, mean; error bars, s.d. Student’s *t* test (two-sided) was used for statistical analysis. **c** Survival rate of mice that were lethally irradiated and then reconstituted with cells derived from mice with the indicated genotypes (*n* = 5 for each group). In black, the non-reconstituted control. **d** Representative histology images showing H&E staining of various organs from PRDM15^+/+^ and PRDM15^∆/∆^ mice 2 months after tamoxifen injection (scale is 100 μm). In panels **a** and **d**, log-rank test was used to determine statistical significance.

To further validate this hypothesis, we took advantage of a recently generated *Prdm15* knockout mouse model^26,27^ (European Conditional Mouse Mutagenesis program) (Supplementary Fig. 3A). We first profiled PRDM15 protein expression in adult mouse tissues, showing that it is ubiquitously expressed, at moderate levels, in a number of organs (e.g. spleen, bone marrow, liver, kidneys) (Supplementary Fig. 3B). The *Prdm15* conditional allele contains loxP sites that flank exon 4 (designated as *Prdm15*^*F*^). We utilized a tamoxifen-inducible PRDM15 KO model (*Prdm15*^*F/F*^*;Rosa26Cre-ERT2*—subsequently referred to as *Prdm15*^*F/F*^*;CreER* or ^*F/F*^ for short), to systemically delete PRDM15 in the whole body (referred to as *Prdm15*^*Δ/Δ*^*;CreER* or ^*Δ/Δ*^ for short). We also used *Rosa26;Cre-ERT2* mice as controls to account for potential non-specific Cre-mediated toxicity. Both groups of mice were injected intraperitoneally (IP) with tamoxifen (TAM) when they were 8-week-old in order to activate the Cre recombinase, resulting in highly efficient rates of recombination in almost all tissues tested, with the exception of the brain (Supplementary Fig. 3C). We found that the resultant PRDM15^Δ/Δ^ mice did not have any conspicuous phenotype and their lifespan was slightly, but significantly increased, compared to controls (Fig. 2a). The PRDM15^+/+^ and PRDM15^Δ/Δ^ mice exhibited comparable weights (Supplementary Fig. 3D) and organ sizes (Supplementary Fig. 3E), and histological analysis did not reveal any overt differences between the various tissues harvested (Fig. 2b). This included highly proliferative organs, such as spleen, intestine, skin, or testis (Fig. 2b). Additionally, there were no significant differences between the PRDM15^+/+^ and PRDM15^Δ/Δ^ mice in terms of bone marrow cellularity, WBC, RBC, and platelet counts, at 2 months following tamoxifen administration (Fig. 2c and Supplementary Fig. 3F). Importantly, PRDM15-depleted hematopoietic stem cells were able to fully reconstitute the hematopoietic system of lethally irradiated mice (Fig. 2d).

Overall, these data demonstrate that in adult mice, PRDM15 is dispensable for normal tissue homeostasis and adult stem cell self-renewal, as opposed to its essential functions in embryonic stem cells and during development^26,28^.

### PRDM15 is critical for lymphomagenesis in vivo

**Fig. 3:**
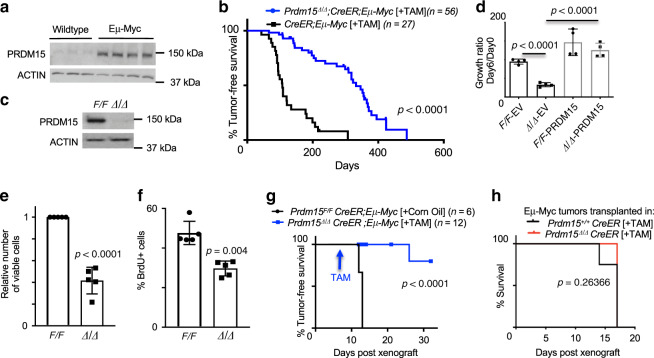
PRDM15 is critical for lymphoma initiation and maintenance in *Eµ*-Myc mice. **a** PRDM15 protein expression in bone marrow-derived B cells from wildtype (*n* = 3) and *Eµ-Myc* (*n* = 4) mice. **b** Disease-free survival of *R26; Eµ-Myc* or *Prdm15*^*F/F*^*; R26; Eµ-Myc* mice injected with TAM at 5 weeks of age. **c** Western blot validation of complete PRDM15 protein depletion upon addition of 4-OHT (OHT) to *Prdm15*^*F/F*^ *Rosa26Cre-ERT2* tumor cells cultured in vitro; data are from a representative experiment (*n* = 20). **d** Growth ratio of *Prdm15*^*F/F*^*;R26; Eµ-Myc* vs. *Prdm15*^∆/∆^*;R26; Eµ-Myc* cells on day 6 post seeding. Cells were treated with 4-OHT (OHT) overnight (day −1) to induce PRDM15 deletion (∆/∆). Prior to the addition of OHT, cells were transfected either with empty vector control (EV) or a vector expressing PRMD15 as indicated. Data are from two independent cell cultures, with two technical replicates each. Center values mean; error bars, s.d. **e** Relative number of viable *Eµ-Myc* cells following PRDM15 depletion by OHT. **f** Relative % of Brdu+ *Eµ-Myc* cells upon EtOH (*Prdm15*^*F/F*^*)* vs. OHT (*Prdm15*^∆/∆^*)* treatment. In **e** and **f**, data are from independent primary lines (*n* = 5). Center values, mean; error bars, s.d. **g** Overall tumor-free survival of mice transplanted with *Prdm15*^*F/F*^*;CreER; Eµ-Myc* lymphoma cells, then injected with corn oil or TAM 1 week later. **h** Overall tumor-free survival of mice transplanted with *Eµ-Myc* lymphoma cells. The background of the recipient mice was either WT (*Prdm15*^*+/+*^*;CreER*) (*n* = 4) or *Prdm15* null (*Prdm15*^*F/F*^*;CreER*) (*n* = 5). Recipient mice were systemically depleted of PRDM15, using TAM, 2 weeks prior to reconstitution with lymphoma cells. In panel **d**–**f**, Student’s *t* test (two-sided) was used for statistical analysis. In panels **b**, **g**, and **h**, log-rank test was used to determine statistical significance.

Given the relevance of PRDM15 in human B-cell lymphomas, we next decided to model disease initiation and progression in the mouse using the *Eµ-Myc* mouse model of B-cell lymphoma^29^. Tumors arising in the *Eµ-Myc* model exhibit pronounced heterogeneity, covering the spectrum between BL and DLBCL^30,31^. We found that PRDM15 was upregulated in bone marrow B cells obtained from *Eµ-Myc* mice (Fig. 3a), suggesting that MYC hyperactivation leads to increase in PRDM15 levels and thus a potential role of PRDM15 in MYC-driven lymphomagenesis. To test this possibility, we crossed *CreER* control mice and *Prdm15*^*F/F*^*;CreER* mice onto the Eµ-Myc background to obtain *CreER;Eµ-Myc* and *Prdm15*^*F/F*^*;CreER;Eµ-Myc* mice. These transgenic mice develop B-cell lymphomas driven by MYC, whereby PRDM15 can be conditionally deleted at various stages of tumor development. To assess the role of PRDM15 in tumor initiation, pretumoral (5-week-old) *CreER;Eµ-Myc* and *PRDM15*^*F/F*^*;CreER;Eµ-Myc* mice were injected with Tamoxifen (TAM) and monitored for disease-free survival. We observed a striking delay in disease onset, whereby the *Prdm15*^*Δ/Δ*^*;CreER;Eµ-Myc* mice showed a median disease-free survival of 332 days, compared to 107 days in control *Eµ-Myc* mice (Fig. 3b). Interestingly, out of 16 tumors isolated from TAM-injected mice, only 5 were B-cell lymphomas (B220+), out of which 4 were escapees (2 expressed PRDM15 in the nucleus, 2 expressed PRDM15 in the cytoplasm) and only one had undetectable levels of PRDM15. The remaining 11 tumors were: 6T cell tumors (CD3+) out of which 4 expressed PRDM15 in the cytoplasm and 2 had undetectable levels of PRDM15, while 5 could not be classified (non-B non-T cell tumors, B220−/CD3−). Taken together, these results provide strong evidence that PRDM15 is critical for B-cell lymphomagenesis (Supplementary Fig. 4A and Supplementary Data 1).

In light of these results, we next isolated primary tumoral B cells from *Prdm15*^*F/F*^*;CreER;Eµ-Myc* mice to study the molecular underpinnings of this phenotype. Acute depletion of PRDM15 by addition of 4-OHT to cell culture media (Fig. 3c) resulted in a severe growth delay of the lymphoma cells, which was rescued by exogenous expression of PRDM15 in the *Prdm15*^*Δ/Δ*^ cells, confirming that this phenotype is caused by PRDM15 loss (Fig. 3d). The reduction in number of viable cells (Fig. 3e) was associated with a decrease in DNA synthesis/proliferation (reduction in the number of BrdU-positive cells) (Fig. 3f), as well as an increase in the number of cells in G1 and G2/M (Supplementary Fig. 4B). Furthermore, PRDM15 depletion caused a significant increase in caspase-dependent apoptosis (Supplementary Fig. 4C).

Next, to assess the role of PRDM15 in tumor maintenance, primary tumors were isolated from tumor-bearing *PRDM15*^*F/F*^*;CreER;Eµ-Myc* mice and transplanted into syngeneic recipient mice. At 7 days post-transplantation, the recipient mice were injected with either corn oil (vehicle control) or TAM to deplete PRDM15. All control mice, but none of the TAM-treated mice, had palpable tumors by day 12, suggesting a cell autonomous function of PRDM15 (Fig. 3g). At this stage (day 12), we sacrificed 6 mice from both groups since all controls had reached the humane end point. Mice with PRDM15-depleted tumors had a significantly lower disease burden than the control group, as determined by the weight of spleen and lymph node (Supplementary Fig. 4D). By FACS analysis, we confirmed that the bone marrow, spleens, and lymph nodes of the TAM-injected mice had a lower percentage of B cells (B220^+^) and that these cells were significantly less proliferative (BrdU^+^) relative to those isolated from control mice (Supplementary Fig. 4E). To corroborate the cell autonomous function of PRDM15 in lymphoma cells, we additionally transplanted *Eµ-Myc* lymphomas in WT or PRDM15 KO recipient mice (*Prdm15*^*F/F*^*;CreER* treated with IP TAM injections; as in Fig. 2a). No difference in disease onset was observed, thus confirming that depletion of PRDM15 in the lymphoma cells, rather than in the tumor microenvironment, is crucial for attenuation of lymphomagenesis (Fig. 3h).

### PRDM15 controls a metabolic program in B-cell lymphoma

**Fig. 4:**
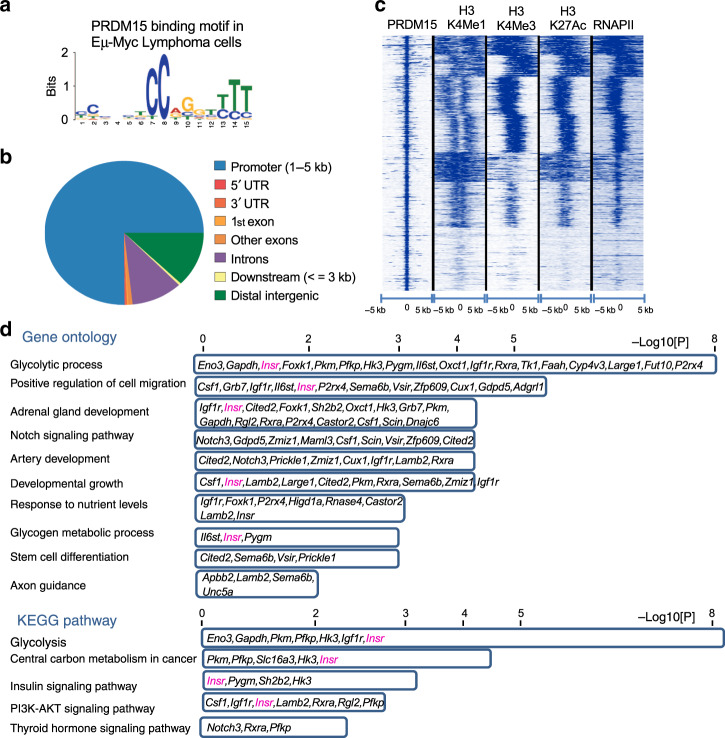
PRDM15 promotes the transcription of key regulators of cancer metabolism. **a** De novo motif discovery. Motif bound by PRDM15 in vivo in *Eµ-Myc* B-cell lymphomas. **b** Distribution of PRDM15 peaks in relation to genes. **c** Density plot of H3K4Me1, H3K4Me3, H3K27Ac, and RNAPII occupancy relative to PRDM15-binding sites in *Eµ-Myc* B-cell lymphomas. **d** Enriched pathways in genes directly bound by PRDM15 and deregulated in *Prdm15*^∆/∆^ *cells*; *Insr* was present in most of the KEGG and GO categories.

To gain further mechanistic insights into the role played by PRDM15 in lymphomagenesis, we proceeded to identify the PRDM15 cistrome. Our previous analysis in ES cells^26^ and in E6.5 embryos^28^ uncovered a specific motif (CC-xxx-TCC-xGx-T/C-T/C-T/C) bound by PRDM15 through its C-terminal zinc fingers. We therefore determined the binding repertoire of PRDM15 in tumoral *Eµ-Myc* lymphoma cells by performing chromatin immunoprecipitation, followed by next generation sequencing (ChIP-seq) and identified 1327 PRDM15 target sites (Supplementary Data 2). De novo motif analysis identified a binding motif identical to that found in mES cells^26^ (Fig. 4a). PRDM15 primarily binds around the TSS of target genes (Fig. 4b), while overlapping with active promoter marks (H3K4Me3/H3K27Ac/RNAPII) (Fig. 4c).

We then analyzed the transcriptome of *PRDM15*^*F/F*^*;CreER;Eµ-Myc* cells before and after PRDM15 depletion induced by 4-OHT treatment in three independent tumors isolated from three different mice (Supplementary Data 3). PRDM15 depletion led to the upregulation of 658 transcripts (Supplementary Data 4), which are mainly involved in regulation of inflammation and cell migration (Supplementary Data 5). On the other hand, 300 transcripts were downregulated upon PRDM15 depletion (Supplementary Data 6). These mRNAs were highly enriched in those encoding metabolic regulators (Supplementary Data 7).

Given the global nature of these metabolic defects, we focused on genes that are directly bound by PRDM15 at their promoters to tease out the initial events, driven by PRDM15, from the secondary ones. Interestingly, we found that many of the direct PRDM15 transcriptional targets (Supplementary Data 8) encoded key regulators of: (i) Insulin/IGF signaling—e.g. *Insr* and *Igf1r* and (ii) glycolysis—e.g. *Pkm, Pfkp, Slc16a3, Eno3, Gapdh,* and *Hk3* (Fig. 4d and Supplementary Data 9).

Indeed, we have validated PRDM15 occupancy at the promoter of several of these metabolic genes (Supplementary Fig. 5A), as well as the transcriptional downregulation induced by PRDM15 loss (Supplementary Fig. 5B). Importantly, these transcriptional changes were also validated in a human ABC-DLCBL line (OCILY3) using a PRDM15-depleting AON (Supplementary Fig. 5C).

### PRDM15 is required for PI3K/AKT/mTOR pathway activity

To establish a role of PRDM15 as a regulator of the INSR/IGF1R/PI3K/AKT/mTOR signaling axis, we first compared the response of several *Prdm15*^*F/F*^*;CreER;Eµ-Myc* cells to PRDM15 depletion and to pharmacological inhibition of the pathway using linsitinib/OSI-906 or everolimus, small-molecule kinase inhibitors (KI) targeting IGF-1R/insulin receptor (IR) and mTORC1, respectively^32^. The effects of both linsitinib and everolimus on cell viability mirrored those observed upon PRDM15 abrogation, as we observed a clear correlation between the sensitivity of primary mouse lymphoma cells and PRDM15 status in the cell for each drug (Supplementary Fig. 6A, B).

**Fig. 5:**
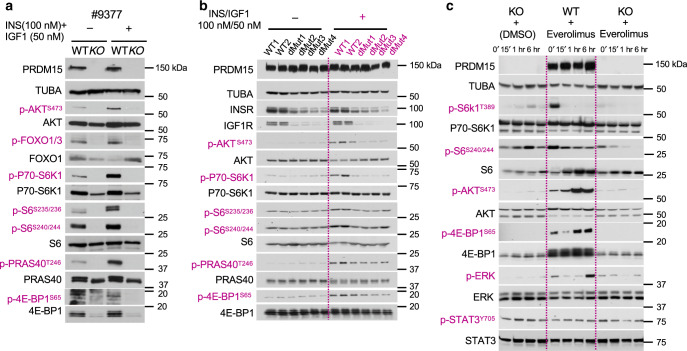
PRDM15 is critical for PI3K/AKT/mTOR activity in *Eµ-Myc* B-cell lymphomas. **a** Western Blot analysis of key signaling axes of the PI3K/AKT/mTOR pathway in starved *Prdm15*^*F/F*^ vs. *Prdm15*^∆/∆^ *Eµ-Myc* B-cell lymphomas, 15’ following stimulation with INSULIN/IGF1. **b** Assessment of PI3K/AKT/mTOR pathway activity, as in **a**, in wild type vs. *Insr*^BSKO^/*Igf1r*^KO^ double mutant *Eµ-Myc* B-cell lymphoma clones. **c** Time course analysis, by WB, of key components of the PI3K/AKT/mTOR to monitor the compensatory phosphorylation/activation of alternative signaling cascades in *Eµ-Myc* lymphoma cells treated with everolimus (mTORC1-i) or/and depleted of PRDM15; *Prdm15*^∆/∆^ cells fail to rewire their signaling. p-STAT3 levels were used as a negative control to show that PRDM15-mediated effects are specific to the PI3K/AKT/mTOR pathway. Data in **a** (*n* = 3 independent primary lines) and **c** (*n* = 2 independent primary lines) are from a representative experiment. Data in **b** are from independent clones (two WT and four mutants).

Next, to validate downregulation of the PI3K/AKT/mTOR pathway in *Eµ-*Myc lymphomas upon PRDM15 depletion, we starved WT and KO cells overnight and subsequently stimulated them with insulin and IGF1. While insulin and IGF1 activated the PI3K/AKT/mTOR pathway in WT cells, as illustrated by increased phosphorylation of AKT, FOXO1/3, P70 S6K, ribosomal protein S6, eukaryotic translation initiation factor 4E (eIF4E)-binding protein 1 (4E-BP1), and the proline-rich Akt substrate of 40 kDa (PRAS40), this response was attenuated in PRDM15 null cells (Fig. 5a).

Transcriptomic data indicated that the upstream regulators of the PI3K/AKT/mTOR pathway, *Igf1R* and *InsR*, are direct transcriptional PRDM15-targets (Fig. 4d). Loss of PRDM15 was associated with reduced chromatin accessibility at the *Igf1R* and *InsR* promoters (Supplementary Fig. 6C) and subsequent downregulation of transcript levels, which were rescued by ectopic expression of PRDM15 (Supplementary Fig. 6d).

To validate this, we induced minimal disruption of the PRDM15-binding site by CRISPR/Cas9 on the promoter region of *Insr* (*Insr*^*BSKO*^) (Supplementary Fig. 7A), which was sufficient to dampen *Insr* expression (Supplementary Fig. 7B–D), followed by CRISPR/Cas9-mediated deletion of *Igf1r*. As expected, this led to downregulation of the AKT/mTOR pathway activity (Fig. 5b). Phenotypically, cells with suppressed *Insr* or *Insr/Igf1r* expression, have a reduced viability, which is however bypassed at later time points (Supplementary Fig. 7E, F). This is consistent with the frequent development of adaptive resistance observed in the clinic in response to linsitinib/OSI-906 and everolimus^33–35^. Indeed, tumor cells acquire resistance to mTOR inhibitors by rewiring their signaling to activate compensatory pathways that enable survival under selective pressure^36,37^. We here show that prolonged, everolimus-mediated, inhibition of mTORC1 in B-cell lymphomas results in disruption of negative feedbacks, thus leading to the hyperactivation of alternative proliferative pathways (i.e AKT and MAPK/ERK) (Fig. 5c). In contrast, PRDM15 depletion alone attenuated mTORC1 signaling without activation of these compensatory pathways, consistent with the almost complete eradication of B-cell lymphomas observed in vivo (Fig. 3b and Supplementary Fig. 4A). The unchanged levels of p-STAT3 in PRDM15 KO cells support the specific effects of *Prdm15* deletion on the PI3K/AKT/mTOR signaling, and not a global downregulation of protein phosphorylation caused by loss of cell viability (Fig. 5c).

Excitingly, from a therapeutic standpoint, our data demonstrate that, while genetic disruption of *Prdm15* has similar effects to the inhibition of *Igf1R/InsR* (linsitinib) and mTORC1 (everolimus), PRDM15 null cells fail to activate alternative proliferative pathways, leading to a more efficient and durable killing of B-cell lymphomas.

### PRDM15 regulates central carbon metabolism in B-cell lymphomas

Recent studies have shown that mTOR inhibition is not effective as monotherapy; several cancer types bypass this inhibition by upregulating glycolysis, while suppression of glycolysis is bypassed by hyperactivation of mTOR and subsequent metabolic reprogramming^38–40^.

**Fig. 6:**
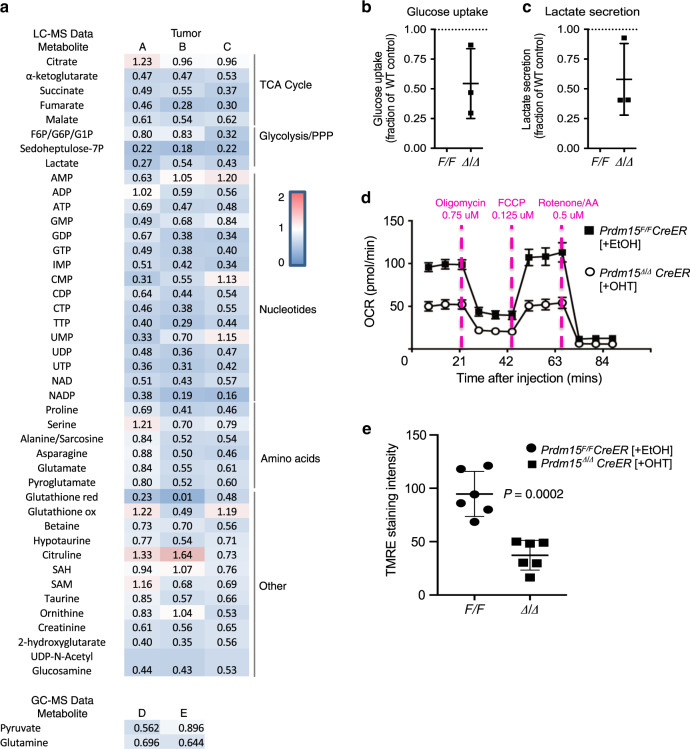
PRDM15 transcriptionally rewires B cell metabolism. **a** Top: Summary of the LC/MS metabolomics analysis in *Prdm15*^*F/F*^ vs. *Prdm15*^∆/∆^ *Eµ-Myc* cells. TCA cycle, glycolysis/pentose phosphate pathway (PPP), nucleotides, amino acids, and other metabolites. Indicated values correspond to the average level of each metabolite in *Eµ-Myc*; *Prdm15*^∆/∆^ cells relative to the *Eµ-Myc*; *Prdm15*^*F/F*^ control cells. Data were collected from three independent tumors (A, B and C), each consisting of three technical replicates. Bottom**:** Summary of the GC/MS metabolite analysis. Pyruvate and Glutamate levels were measured in two independent tumors. The average is shown for tumors D and E. Data represents three biological replicates, each performed in technical triplicates. **b** and **c** One day post PRDM15 KO-induction, *Prdm15*^*F/F*^ (WT) and *Prdm15*^∆/∆^ cells were seeded in fresh media. 24 h later, glucose and lactate concentration in the cellular media were measured and subtracted from that of media alone. **b** Glucose uptake and **c** lactate secretion are shown relative to the *Prdm15*^*F/F*^ (WT) cells. Error bars represent SD (*n* = 3 primary lines). **d** Seahorse Mito Stress Test Assay. *Prdm15*^*F/F*^*;CreER;Eµ-Myc* tumor cells were treated with either EtOH (control) or OHT (to induce PRDM15 depletion). Cells were then seeded onto 96-well Seahorse assay plates to measure cellular respiration. Compounds were loaded into a hydrated cartridge in the following order: Port A 25 µl Medium, Port B 0.75 µM Oligomycin, Port C 0.125 µM FCCP, and Port D 0.5 µM Rotenone/AntimycinA. **e** TMRE staining. *Prdm15*^*F/F*^*;CreER;Eµ-Myc* tumor cells were treated with either EtOH (control) or OHT (to induce PRDM15 depletion) and stained with TMRE at concentration of 200 nM for 30 min before washing off. Data are from independent primary lines (*n* = 6). Center values mean; error bars, s.d. Cells were imaged and quantified using ImageJ analysis software (*n* =3  images/sample analyzed). Student’s *t* test (two-sided) was used for statistical analysis.

Our transcriptomic data indicate that PRDM15 controls a transcriptional program linked to central carbon metabolism, by regulating the expression of key glycolytic genes (e.g. *Pkm, Pfkp, Slc16a3, Eno3, Gapdh*, and *Hk3)*. This prompted us to investigate the effects of PRDM15 depletion on the metabolome of B lymphoma cells. For this purpose, *Prdm15*^*F/F*^;CreER;Eµ-Myc, treated with EtOH or 4-OHT, were collected for metabolome analysis by LC/MS and GC/MS. To capture the early events induced by PRDM15 depletion, cells were collected as soon as PRDM15 was completely depleted (48 h after addition of 4-OHT). Of note, neither cell viability nor cell size were significantly affected by PRDM15 loss at this time point (Supplementary Fig. 8A, B). We detected broad alterations in the metabolome of PRDM15 WT vs. KO cells, which corresponded to the observed transcriptional reprograming (Fig. 6a). The metabolome data was normalized over viable cell population count, since we did not observe any major differences in cell viability and/or size between WT and PRDM15 KO cells (Supplementary Fig. 8A, B). In accordance with the reduction in the expression of genes involved in glucose metabolism, we detected a substantial reduction in glycolytic intermediates (i.e. F6P/G6P/G1P) and lactate in PRDM15 depleted vs. control cells (Fig. 6a). This was accompanied by a decrease in glucose uptake upon PRDM15-depletion (Fig. 6b), which is consistent with the downregulation of the *Slc2a1* (*Glut1*) transporter and the decrease in lactate secretion (Fig. 6c). In addition, we observed that the levels of the pentose phosphate pathway (PPP) intermediate sedoheptulose-7P were significantly reduced upon PRDM15 loss, consistent with the downregulation of PPP enzymes (*Gpi, Aldo, Pfkp, Pfkm, Pfkl*). The PPP is required for the de novo synthesis of nucleotides. Accordingly, and in keeping with the downregulation of genes playing a role in purine and pyrimidine synthesis (*Tk1*), levels of IMP and NTPs were lower in cells devoid of PRDM15 relative to controls (Fig. 6a). Loss of PRDM15 also resulted in decrease in citric acid cycle (CAC) intermediates including α-ketoglutarate, malate, succinate, and fumarate, as well as a number of amino acids including asparagine, glutamate, and proline (Fig. 6a). Intriguingly, levels of citrate, serine, and some additional metabolites remained comparable between PRDM15-proficient and deficient cells (Fig. 6a), which together with the absence of dramatic differences in cell viability and size at the investigated timepoints argues against that the effects of PRDM15 depletion on metabolome are secondary to advanced cell death. In support of this point, the dependency of β-oxidation to fuel cellular respiration is also comparable between PRDM15-proficient and deficient cells (Supplementary Fig. 8C). Collectively, these results indicate that the loss of PRDM15 has a profound impact on the B-cell lymphoma metabolome, which matches its effects on the transcriptome (Supplementary Fig. 5A–C).

The upregulation of glutamine uptake and utilization is thought to be a hallmark of MYC-driven tumors, including B-cell lymphomas^41–43^. Indeed, glutamine plays a central role in cancer cell metabolism, as it represents an important source of nitrogen for the synthesis of key cellular metabolites (e.g. nucleotides, amino-acids, glutathione, lipids), while additionally providing an anaplerotic source of carbon for the CAC^44,45^. We confirmed this in our model by performing isotope-tracing experiments wherein *Prdm15*^*F/F*^*;CreER;Eµ-Myc* tumor cells were labeled with ^13^C_5_-glutamine. Consistent with previous reports on MYC-driven tumors, CAC cycle intermediates α-ketoglutarate m+5, malate m+4, fumarate m+4, citrate m+4) incorporated carbon ^13^C at rates of 40–50% following 3 h incubation with ^13^C_5_-glutamine, which supports that they utilize glutamine as an anaplerotic source to replenish CAC (Supplementary Fig. 8D). Accordingly, glutamine depletion strongly suppressed proliferation of *Prdm15*^*F/F*^*;CreER;Eµ-Myc* cells, indicating that they have become dependent on glutamine (Supplementary Fig. 8E). Considering that the loss of PRDM15 debilitated glucose uptake and utilization, we next investigated whether PRDM15 may influence anaplerotic utilization of glutamine. Analysis of glutamine levels in the media suggested that PRDM15-loss resulted in decreased glutamine utilization (Supplementary Fig. 8F). To this end, we performed stable isotope tracer analysis (SITA) using ^13^C_5_-glutamine in WT vs. PRDM15 KO cells, and we investigated both (i) the fraction of ^13^C-labeled metabolites relative to the total pool and (ii) the total ^13^C-labeled amount of CAC cycle intermediates. Although the total ^13^C-labeled CAC intermediates levels were decreased upon PRDM15 KO (fumarate, malate, succinate, α-ketoglutarate; Supplementary Fig. 8G), in both WT and PRDM15 KO cells the fraction of ^13^C-labeled CAC cycle metabolites is largely unaffected by the PRDM15 status (Supplementary Fig. 8H). Hence, the decrease in total ^13^C-labeled metabolites upon PRDM15 KO appears to largely stem from the decrease in total metabolite levels, but not from the proportional tracing of glutamine through the CAC. This suggests that the activity of the CAC enzymes is not affected by the PRDM15 KO, and that the production of CAC cycle intermediates is decreased due to a reduction in glutamine uptake and/or utilization (Supplementary Fig. 8F). Overall, these findings argue that in addition to impairing glucose metabolism, PRDM15 also abrogates glutamine utilization, without exerting a major effect on the activity of CAC enzymes.

Consistent with these findings, PRDM15 loss resulted in decreased oxygen consumption rates (OCR) (Fig. 6d), which together with the observed reduction in metabolites indicates that the depletion of PRDM15 impedes the respiratory and glycolytic capacity of lymphoma cells. In addition, we measured the mitochondrial membrane potential by TMRE staining and confirmed the reduction of mitochondrial membrane potential in cells depleted for PRDM15 (Fig. 6e and Supplementary Fig. 8I). Altogether, these results are consistent with a model whereby loss of PRDM15 in B-cell lymphomas results in a transcriptional downregulation of key metabolic genes causing a metabolic crisis which then leads to cell death.

## Discussion

We here identify PRDM15 as a transcription factor that is broadly dispensable in adult homeostatic functions but has critical roles in B-cell lymphomagenesis. Depletion of PRDM15, genetically or using antisense drugs, impairs tumor growth and induces specific killing of B-cell lymphomas, both in vitro and in vivo. In lymphoma cells, PRDM15 regulates the transcription of key genes involved in two major metabolic axes required for tumor cell survival: PI3K/AKT/mTOR signaling (*InsR* and *Igf1R*) and glycolysis (e.g *Pkm, Pfkp, Eno3)*.

A substantial crosstalk exists between mTOR signaling and glycolysis, which often leads to activation of compensatory mechanisms to bypass inhibition of one axis or the other^38–40^. Combined inhibition of these axes was suggested to provide efficient therapeutic strategy in several cancer types. Our data show that, besides downregulation of glycolysis, PRDM15 depletion is sufficient to drive a sustained inhibition of mTOR signaling, as opposed to everolimus or linsitinib as single agents. Perturbation of both processes results in profound and broad metabolic defects, as indicated by reduction in glycolytic and TCA cycle intermediates, nucleotides and most of amino acids content, leading to a metabolic crisis and cell death.

Our transcriptomic data indicate that besides the mentioned PI3K/AKT/mTOR and glycolytic genes, PRDM15 regulates key regulators of other metabolic processes as well as genes involved in NOTCH and WNT signaling (i.e. *Tk1, Faah, Notch3*, and *Prickle1*, etc.) (Fig. 4d). The relevance of these additional targets, as well as potential mechanisms of how PRDM15 controls uptake and/or utilization of glutamine in the context of lymphoma warrants further investigations. Of note, while acute reduction of *Igf1R/InsR* expression was able to mimic in the short term, PRDM15 depletion—before cells are able to rewire— the overexpression of either or both *Igf1R/InsR*, was only partially able to rescue the complex phenotype induced by PRDM15 depletion. Partial rescue was observed in certain, but not all clones, underscoring the broad effect of PRDM15 on multiple metabolic pathways, including the PI3K/AKT/mTOR axis, among others.

Several recent studies suggested that metabolic plasticity of cancer cells limits therapeutic approaches aiming to target oncogenic metabolic programs^46,47^. Overall, our data indicate that PRDM15 regulates an entire transcriptional program governing several key metabolic pathways. We reason that the global transcriptional regulation controlled by PRDM15 makes it a very attractive target in oncology, as our results indicate that PRDM15 KO cells fail to rewire their metabolic programs or to develop resistance to PRDM15 depletion (Fig. 5c). Indeed, this is what we observe in the in vivo mouse models where we have a substantial delay in lymphomagenesis (Fig. 3b), and mice succumb rarely to B-cell tumors (when tumors escape *Prdm15* deletion), but rather to late onset T and non-B/T malignancies (Supplementary Fig. 4A and Supplementary Data 1). Moreover, the low levels of PRDM15 expression in multiple organs and its apparent dispensability in normal adult tissues, provide a wide therapeutic window.

To conclude, we here uncover a PRDM15-driven transcriptional program, pertinent to the high energy demands of B-cell lymphomas. We propose that targeting PRDM15 in these tumors concurrently inhibits PI3K/AKT/mTOR and glycolysis, which importantly are pathways not regulated by PRDM15 in ES cells or during development^26,28^. This dual blockade leads to a metabolic crisis that cannot be tolerated by lymphoma cells, which holds a promise to resolve the issues related to acquired resistance and metabolic rewiring associated with the current therapies targeting the PI3K/AKT/mTOR pathway. More importantly, our protein expression analysis and in vivo functional assays support a wide therapeutic window and suggest that PRDM15 would be an excellent target for B-cell lymphoma treatment.

## Methods

### Animal protocols

All mouse experimental protocols are approved by the Institutional Animal Care and Use Committee (IACUC), A*STAR. The animals were maintained according to the specified standard of care, in compliance with international guidelines. Mice were monitored daily for any sign of distress and the development of palpable lymph nodes (tumors).

### Mouse strains and genotyping

The *Prdm15*^*F/F*^*;Rosa26Cre-ERT2* mice have been described in details^26^. Eµ-Myc mice were purchased from Jackson Labs. Briefly, tails were digested in lysis buffer (100 mM sodium chloride, 25 mM EDTA, 10 mM Tris buffer (pH 8.0), 0.5% SDS, 0.2 mg/ml Proteinase K). Following incubation at 50 °C overnight, samples were centrifuged, and the supernatant was mixed with 2-propanol (Isopropanol) in a 1:1 ratio. The precipitated DNA was pelleted, dried, and resuspended in Tris-buffer (pH 8.0).

100 ng of template DNA, 500 nM primers, and DreamTaq Green PCR master mix (Thermo Scientific #K10820) were combined for PCR. All primers are listed in Supplementary Data 10. The following cycling conditions were used: 95 °C for 5 min; 34 cycles of 95 °C for 45 s, 60 °C for 30 s and 72 °C for 40 s; 72 °C for 5 min.

### Mouse tissue collection

Whole blood was collected from the tail vein of mice using heparinised microhaematocrit capillary tubes (Fisher #22-362-566), and analyzed using Hemavet 950FS. For tissue collection, the mice were sacrificed according to the IACUC guidelines, and tissues were collected for subsequent analysis.

### Histology and immunohistochemistry (IHC)

Tissues were fixed in 4% paraformaldehyde (PFA) for 72 h and embedded in paraffin blocks (Thermo Scientific Excelsior AS Tissue Processor and HistoStar Embedding Workstation) and microtomed onto glass slides at 5 µm. The sections were then either stained with hematoxylin and eosin (H&E), or immunohistochemically for CC3 and Ki67 Leica Bond-Max^TM^ auto-stainer. Quantification of the necrotic area was performed based on examination of a single H&E-stained section of each tumor. Counterstaining for CC3 and Ki67 were also used to evaluate and distinguish between necrotic and apoptotic or proliferating areas, respectively. This work was performed in collaboration with the Advanced Molecular pathology Lab (AMPL) in IMCB.

### Quantitative analysis of IHC images

The TMA slides were imaged using the Multispectral Vectra 2 Imaging System (PerkinElmer Inc., Waltham, MA, USA). Multispectral images were analyzed using the inForm 2.2 software (PerkinElmer Inc., Waltham, MA, USA). To obtain monochrome images of both 3,3′-diaminobenzidine (DAB) and haematoxylin components of the IHC slide, images were first unmixed using a prepared spectral library of dye-specific pure spectra measured from single-stained slides of each dye. A trainable tissue segmentation algorithm was used to identify regions of interest containing tumor cells; and correct tissue segmentation was reviewed by a pathologist. Germinal centre and non-germinal centre regions were annotated manually in tonsil tissue. Cells were segmented using a cell segmentation algorithm, creating a haematoxylin-based nuclear mask for each cell within the tumor region. PRDM15 expression per patient was expressed as mean nuclear optical density (OD, peak weighting) of DAB staining.

### Cell culture

Primary Eμ-Myc lymphoma and bone marrow cells were isolated and maintained in the appropriate culture medium^25^. OCI-LY3, MC116, Karpas 231, HT, and PR1 cells were a kind gift from Dr. Ong Sin Tiong (Duke NUS) and originally purchased from ATCC. OCI-LY3 and MC116 cells were maintained in RPMI 1640 supplemented with 20% FBS. P493, Karpas 231, HT, and PR1 cells were maintained in RPMI 1640 supplemented with 10% FBS. All cells were grown in a humidified incubator at 37 °C and 5% CO_2_.

### AON design

AONs were rationally designed for optimal efficiency in inducing splicing modulation. Briefly, the AON target sites were selected by a computational algorithm that accounts for co-transcriptional-binding accessibilities, binding thermodynamics, and the presence of regulatory splicing motifs. The AONs were synthesized by Sigma Aldrich (Singapore) as single-stranded 2′-O-methyl-modified RNA bases linked with phophorothioate backbone. The AON sequences are as follows:

PRDM15: ACU CAC AGG CUC AUC CGG AGG GAC

Scrambled: CCU UCC CUG AAG GUU CCU CC 

### AON transfection protocol

Cells were transiently transfected with AONs using the Neon Transfection system (Invitrogen). Briefly, the cells were resuspended in R buffer at a density of 1e7 cells/ml with 10 µM AON. 10 µl of sample was electroporated at 1300 V × 20 s × 2 pulses, plated into 2 ml fresh media.

### DLBCL PDX xenograft construction and treatment

Tumor fragments were implanted subcutaneously into the flanks of 4–6-week-old female NOD/SCID mice. When the xenografts reached 150–200 mm^3^, the mice were randomized into two groups (each *n* = 8), for treatment with Scrambled or PRDM15-targeting AON. The AONs were prepared at 3 mg/ml in PBS, and 100 µl was injected intra-tumorally every other day for 20 days (i.e. 10 doses). On Day 21, the mice were sacrificed, and the tumors were collected.

### Induction of PRDM15 deletion in vitro and in vivo

In all in vitro experiments, *Prdm15*^*F/F*^;*ROSA26-CreER* cells were treated with either 50 nM OHT (Sigma H7904) or equal volume of ethanol for 24 h. Cells were then washed and resuspended in fresh media. For in vivo experiments, 2 mg tamoxifen (Sigma T5648) was administered intraperitoneally in adult mice for 3 consecutive days. In experiments with *Prdm15*^*F/F*^; ROSA26-CreERT2 animals, we used R*OSA26:CreER* counterparts as negative controls, ensuring that the addition of OHT or tamoxifen (TAM) was not toxic^48^. The doses of tamoxifen and 4-OHT were carefully titrated to the minimum necessary for effective recombination, while minimizing side effects.

### Recombination PCR

To confirm the deletion of the *Prdm15* floxed exon (exon 4) following activation of Cre-recombinase, genomic DNA from cells was isolated using the Qiagen DNeasy blood & Tissue kit (cat. No. 69506). DNA was isolated from mouse tissues according to the genotyping protocol described above. The following primers were used for amplification:

Fwd-AAGACATTGGGTGCACAG, Rev-GGCTTCTGGGGTTCACTTT. The following cycling conditions were used for PCRs: an initial incubation at 95 °C for 5 min, followed by 36 cycles of denaturation (45 s at 95 °C), annealing (30 s at 57 °C), elongation (2 min at 72 °C), and a terminal incubation at 72 °C for 7 min.

### 5-Bromodeoxyuridine (BrdU) labeling and flow cytometry analysis

Cells were labeled with 50 μM BrdU for 1 h, after which they were collected and fixed in ice cold 70% ethanol O/N at −20 °C. The fixed cells were then incubated in DNA denaturation solution (2 M hydrochloric acid, 0.1% Triton X-100) for 30 min, followed by neutralization with 0.1 M sodium tetraborate, pH 9.0. Subsequently, cells were stained with 5 µl BrdU-FITC antibody (Becton Dickinson 7583) diluted with 25 µl FACS buffer (PBS, 0.5% Tween 20, 1% BSA) for 30 min at room temperature, protected from light. The cells were washed once with FACS buffer and resuspended in DNA staining solution (20 μg/ml propidium iodide (PI), 0.1% Triton X-100, and 0.25 mg/ml RNAse A in PBS). The samples were incubated for 30 min at RT, before being analyzed by flow cytometry.

### Cell viability and apoptosis assays

Cell viability was assessed using CellTiter-Glo Luminescent Cell Viability Assay (Promega G7570) or using trypan blue exclusion, and apoptosis was assessed using Caspase-Glo 3/7 Assay (Promega G8091). Both were used according to the manufacturer’s protocol. All assays were performed in opaque, white, flat-bottomed 96-well plates (Corning), and luminescence was measured on a Tecan Safire2 plate reader.

### Quantitative real time PCR (qRT-PCR)

Total RNA from cells was isolated using PureLink RNA Mini Kit (Ambion-1283-018A), according to the manufacturer’s instructions, and quantified on a Nanodrop 2000 (Thermo Scientific). For tissue samples, single cell suspensions were generated by pressing the tissues through a 40 μm mesh strainer. The cells were lysed in TRIzol reagent, and RNA was extracted according to the “Using TRIzol reagent with the PureLink RNA Mini Kit” protocol provided by the manufacturer. 2 μg of RNA was retro-transcribed into cDNA using Maxima First Strand cDNA Synthesis Kit (Thermo Scientific K1642), and subjected to qRT-PCR on an ABI PRISM 7500 machine. PCR reactions (20 μl) contained 10 μl SYBR Green PCR supermix (2×), 4 μl of a forward and reverse primer mix (final concentration 200 nM) and 6 μl cDNA (20 ng). All primers were designed using Primer3 software (version 0.4.0). The sequences of the primers used are listed in Supplementary Data 10.

### Western blotting

Cells were lysed in 1X Laemmli buffer, sonicated (3 × 15 s), and boiled for 5 min at 98 °C. Protease/phosphatase inhibitor cocktail (CST-5872s) was added for all experiments involving phosphorylated proteins. Proteins were quantified using the RC DC Protein Assay Kit (Biorad 5000121). 20–50 μg of proteins were loaded onto 6–15% gels and separated by SDS–PAGE. The samples were then transferred to nitrocellulose membranes (Whatman Protran 0.2 μm), which were blocked in either 5% dry milk (Sigma 70133) or BSA (Sigma A7906) in TBST buffer (0.1% Tween 20 in 1X TBS) for 1 h. The membranes were incubated with the indicated primary antibodies (diluted either in 5% dry milk or BSA) overnight at 4 °C. The day after, membranes were washed in TBST, incubated with HRP-conjugated secondary antibodies for 1 h at RT, and washed in TBST. The signals were visualized on X-ray films (Fujifilm 47410 19289) using the SuperSignal West Pico Chemiluminescent Substrate (Thermo Scientific 34080). Details of the antibodies used are in Supplementary Data 12.

### CRISPR-cas9 editing

*Eµ*-Myc B-cell lymphoma lines were transfected with PX458 [pSpCas9 (BB)−2A-GFP] vector expressing a guide RNA targeting the site to be mutated. PRDM15-binding site on INSR was extracted from the ChiP-seq data. Genomic DNA from multiple clones was used for screening by Genescan. Mutant clones were then cloned into the pCR 4-TOPO TA vector following the manufacturer’s instructions and validated by sanger sequencing. Single guide RNA sequences are described in Supplementary Data 11.

### Immunostaining of BM and spleen cells for FACS analysis

Single cell suspension of mice spleen and bone marrow cells were washed with FACS buffer (PBS with 0.5% FBS, 2 mM EDTA) prior to immunostaining. Cells were stained with antibody cocktail of antibodies from Miltenyl Biotec against CD45 (1:100), CD11b (1:100), CD3 (1:100), B220 (1:20), and CD19 (1:20) at a density of 10^6^ cells per 50 μl volume for 30 min on ice. Thereafter, cells were washed two times with FACS buffer and acquired using BD LSR II.

### Chromatin immunoprecipitation (ChIP)

All steps of ChIP experiments were carried at 4 °C in the presence of protease inhibitors, unless stated otherwise. In brief, 50 million cells were fixed in 1% formaldehyde for 10 min at RT. The reaction was quenched by adding glycine to a final concentration of 0.125 M. The cells were washed in PBS, harvested in SDS lysis buffer and frozen at −20 °C overnight. The next day, the cells were pelleted by centrifugation, resuspended in ice-cold IP buffer and sonicated for 4–6 cycles of (30 s ON/60 s OFF) at 30% amplitude using a Branson Digital Sonifier (# S540D) to obtain chromatin fragments of ~300–800 bp. The lysates were precleared for 4 h in a 1:1 A/G Sepharose beads mix (blocked in 5 mg/ml lipid-free BSA). The beads were removed by centrifugation and samples were incubated with PRDM15 antibody with rotation overnight at 4 °C. The next day, the samples were spun-down for 20 min at 300×*g*. Protein A/G Sepharose beads were added, and the samples were incubated with rotation for 4 h at 4 °C. The beads were then pelleted by centrifugation and washed. The immunoprecipitated DNA was eluted in 1% SDS and 0.1 M NaHCO_3_, and the samples were de-crosslinked overnight at 65 °C. The eluted material was purified with QIAquick PCR purification Kit (Qiagen 28104), and the DNA was resuspended in T- buffer (10 mM Tris–HCl, pH 8).

### Chip-sequencing and bioinformatics analysis

For ChIP-sequencing, DNA libraries were prepared using the TruSeq ChIP Sample Prep Kit (IP-202-1012), following the manufacturer’s instructions, and sequenced in the Illumina Hiseq 2000 and Nextseq 500 at the Genome Institute Singapore (GIS). The sequenced reads were mapped to mm9 build of the mouse genome from University of California Santa Cruz (UCSC) genome database using Bowtie 0.12.8^49^ with default parameters except –m 1 and –segment-mismatches 2. Only reads which mapped uniquely to the genome with at most two mismatches were kept. Duplicate reads were filtered by MACS (2.1.0)^50^ to limit PCR-induced biases and the *q*-value was set to 0.01 for peak calling.

Annotations of nearby genes associated with PRDM15 peaks were performed using GREAT (2.02)^51^. Each gene was assigned a basal regulatory domain defined as prompter region (−5 kb/+5 kb), and a distal regulatory region (50 kb) extended in both directions. *K*-means clustering of PRDM15 peaks and histone modifications in mESCs were performed with seqMINER^52^.

Gene ontology analysis of associated genes was performed using Metacore^53^. The associated GO terms were ranked by *p*-value. 200 bp wide DNA sequences of PRDM15 peak regions were used to identify enriched motifs. The enriched motifs were discovered by using the Multiple Em For Motif Elicitations (MEME) software suite (http://meme.nbcr.net/memecgi-bin/meme-chip.cgi)^54^ with default settings. Reported motifs were ranked according to *E*-value significance, which is the estimated probability of the expected number of motifs with the given log-likelihood ratio compared to a random set of sequences of similar size and sequence width. Series records GSE116905 and GSE116906 provide access to all data presented in this manuscript.

### RNA-sequencing and bioinformatics analysis

Library preparation was performed following the TruSeq RNA sample preparation v2 guide (Illumina). In brief, the sequenced reads were mapped to mm9 build of the mouse genome from University of California Santa Cruz (UCSC) genome database using STAR v2.4.2a^55^ with default parameters. Paired differential expression analysis was performed using edgeR with gene model annotations from Ensembl (version NCBIM37.65). Normalization and differential expression were called according to the standard methods implemented by and outlined in the EdgeR package. Genes that are represented at least 1 cpm (counts per million) reads in at least two samples were kept for analysis. Data was fitted to a negative binomial generalized log-linear model to the read counts for each gene. Genes with fold changes >1.5 were considered significantly regulated. Enriched Gene Ontology terms and KEGG pathways were identified using Metacore. Heatmaps of gene expression (FPKM) were generated using the R software and packages (EdgeR version 3.28.1 released under Bioconductor 3.10). Sequencing datasets were deposited into NCBI GEO -GSE116905 and GSE116906.

### Insulin stimulation

To test whether the activity of the Pi3K/AKT/mTOR-signaling pathway is affected by the presence or absence of PRDM15, WT and KO cells were washed several times with PBS to remove residual serum, then starved overnight. The day after, the cells were stimulated with a combination of INS (100 nM) and IGF1 (50 nM) in a serum-free medium for 15 min before collection for protein extraction. To compare the effects of everolimus vs. PRDM15 depletion, WT and KO cells were starved overnight then transferred to complete medium for 15′ to activate signaling pathways (*T* = 0). Cells were then treated with everolimus or vehicle for the indicated timepoints.

### Seahorse mito stress test assay

Cells were seeded onto 96-well seahorse assay plates coated with Cell-Tak (25 µl of 22.4 µg/ml per well) by centrifugation, 200 × *g* for 3 min with zero braking. Assay was performed using Seahorse XF DMEM medium supplemented with 2 mM l-glutamine, 10 mM glucose, and 1 mM sodium pyruvate. Compounds were loaded into a hydrated cartridge in the following order: Port A 25 µl Medium, Port B 0.75 µM Oligomycin, Port C 0.125 µM FCCP, and Port D 0.5 µM Rotenone/Antimycin A.

### TMRE staining

Cells were seeded onto black clear-bottom 96-well plates with Cell-Tak (50 µl of 22.4 µg/ml per well) by centrifugation, 200 × *g* for 3 min with zero braking. Cells were stained with TMRE at concentration of 200 nM for 30 min before washing off. Cells were imaged using Nikon Eclipse Ti microscope and quantification of TMRE staining was achieved using ImageJ analysis.

### Glucose, glutamine consumption, and lactate release assays

Using three independent *Prdm15*^*F/F*^*;CreER;Eµ-Myc* tumor cell lines, PRDM15 depletion was induced by treatment for 1 day with OHT (50 nM) (indicated as ∆/∆), with EtOH as vehicle control (indicated as WT or F/F). The end of the induction period is considered as day 0. The next day, cells were spun down, resuspended in fresh media and plated at 1 or 2.5 million cells/ml in six-well plates. A six-well plate with media alone was incubated in parallel. 24 h later, supernatants were collected, and cells were counted using an automated cell counter (Invitrogen). Measurement of glucose and glutamine concentration in samples was done using a BioProfile 400 analyzer (Nova Biomedical). Total consumption was calculated by subtracting results from baseline glucose or glutamine concentration, measured in samples from media incubated in identical conditions, without cells. Molar concentrations of glucose and glutamine were normalized per 10^6^ cells and normalized relative to WT (F/F) control. Lactate release was quantified using a lactate assay kit (BioVision). Total secretion was calculated by subtracting results baseline lactate concentration, measured in samples from media incubated in identical conditions, without cells. Lactate production was normalized to cell count and data were expressed as fraction of WT (F/F) cells.

### GC/MS and stable isotope tracer analyses

Using three independent *Prdm15*^*F/F*^*;CreER;Eµ-Myc* tumor cell lines, PRDM15 depletion was induced by treatment for 1 day with OHT (50 nM) (indicated as ∆/∆), with EtOH as vehicle control (indicated as WT or F/F). The end of the induction period is considered as day 0. The next day, WT (F/F) and PRDM15 ∆/∆ cells were resuspended in fresh media at 1 million cells/ml for an additional 6 h, then collected on ice for steady-state GC/MS analysis. ^13^C_5_-glutamine stable isotope tracer analyses were performed as in ref. ^46^. One day post-OHT induction, cells were resuspended in glutamine-free fresh media supplemented with 6 mM ^12^C-glutamine for 2 h to equilibrate the cells. Then, cells were spun and resuspended in glutamine-free fresh media, supplemented with 6 mM ^13^C_5_-glutamine (CLM-1822-H-PK; Sigma), for 30, 60, and 180 min, then collected on ice for GC/MS analysis. For GC/MS analysis, cells were rinsed three times with 4 °C saline solution (9 g/L NaCl) and quenched with 600 µl 80% MEOH (<20 °C). Membranes disruption was carried out by sonication at 4 °C (10 min, 30 s on, 30 s off, high setting, Diagenode Bioruptor). Extracts were cleared by centrifugation (13,000 × *g*, 4 °C) and supernatants were dried in a cold trap (Labconco) overnight at −4 °C. Pellets were solubilized in 30 µl pyridine containing methoxyamine–HCl (10 mg/ml, Sigma) by sonication and vortex, centrifuged and pellets were discarded. Samples were incubated for 30 min at 70 °C (methoximation), and then were derivatized with MTBSTFA (70 µl, Sigma) at 70 °C for 1 h. 1 µl was injected per sample for GC–MS analysis. GC–MS instrumentation and software were all from Agilent. Data analyses were performed using the Chemstation software (Agilent, Santa Clara, USA). For steady-state values, data were collected from three independent tumors, each consisting of three technical replicates. For isotope-tracing experiments, data were collected from three independent tumors.

### LC/MS analysis

1 day post OHT induction, WT (F/F) and PRDM15 ∆/∆ cells were collected on ice, washed with 3 ml of ice-cold 150 mM ammonium formate solution (pH: 7.4) three times, followed by addition of 230 µl of 50% MeOH/50% water solution (pre-chilled to −20 °C). Six pre-washed 1.4 mm ceramic beads were added to tubes. This was followed by addition of 220 µl of −20 °C acetonitrile (ACN) and a 10 s vortex. The samples were loaded into pre-chilled bead beater racks (TissueLyser II—Qiagen) and beaten for 2 min at 30 Hz. 600 µl of 4 °C dichloromethane and 300 µl pre-chilled (4 °C) LC/MS water was added. The samples were vortexed for 1 min and allowed to partition on ice for 10 min. The samples were then centrifuged for 10 min at 1750 RCF and 4 °C. The upper aqueous phase was transferred to a new Eppendorf tube on dry ice. Samples were dried at −4 °C using a temperature-controlled vacuum centrifuge (Labconco, Kansas City, MO, USA). The samples were stored at −80 °C until analyzed. Samples were resuspended in 30 µL cold LC/MS grade water and clarified by centrifugation 10 min, 13,000×*g* at 1 °C. Supernatants were transferred to LC/MS vials containing 200 µL Teflon inserts. A volume of 5 µL was injected for each LC–MS/MS analysis. All semi-quantitative LC–MS/MS analyses were carried out on Agilent 6430 Triple Quadrupole (Agilent Technologies, Santa Clara, CA, USA). Chromatography was achieved using a 1290 Infinity ultra-performance LC system (Agilent Technologies, Santa Clara, CA, USA) consisting of vacuum degasser, autosampler and a binary pump. Relative concentrations were determined by integrating area under the curve for the quantifying dMRM transition and compared to external calibration curves. Data were analyzed using MassHunter Quant (Agilent Technologies, Santa Clara, CA, USA). No corrections were made for ion suppression thus data are considered to be semi-quantitative. *Nucleotides*: Nucleotide separation was achieved through the use of a Scherzo SM-C18 column 3 μm, 3.0 × 150 mm (Imtakt Corp., Japan) maintained at 10 °C. The chromatographic gradient started at 100% mobile phase A (5 mM ammonium acetate in water) with a 5 min gradient to 100% B (200 mM ammonium acetate in 20% ACN/80% water) at a flow rate of 0.4 ml/min. This was followed by a 5 min hold time at 100% mobile phase B and a subsequent re-equilibration time (6 min) before next injection. A sample volume of 5 µL of sample was injected for analysis. Sample temperature was maintained at 4 °C before injection. Eluting nucleotides were detected using an electrospray ionization (ESI) source operating in positive ionisation mode. Dynamic multiple reaction monitoring (dMRM) were optimized on authentic standards for each targeted metabolite. Gas temperature and flow were set at 350 °C and 10 l/min, respectively, nebulizer pressure was set at 40 psi and capillary voltage was set at 3500 V. *TCA cycle intermediates, Glycolysis and Pentose Phosphate Pathway* (*PPP*). Metabolite separation was achieved through the use of a Scherzo SM-C18 column 3 μm, 3.0 × 150 mm (Imtakt Corp., Japan) maintained at 10 °C. The chromatographic gradient started at 100% mobile phase A (100 mM formic acid in water) for 2 min post sample injection. The hold was followed with a 6 min gradient to 80% mobile phase B (200 mM ammonium formate in 30% CAN pH = 8)) at a flow rate of 0.4 ml/min. This was followed by a 5 min hold time at 100% mobile phase B and a subsequent re-equilibration time (6 min) before next injection. A sample volume of 5 µL of sample was injected for analysis. Sample temperature was maintained at 4 °C before injection. Eluting compounds were detected by ESI in both positive and negative ionization modes and dMRM optimized on authentic standards. ESI source conditions were the same as for the nucleotide’s analysis.

### Amino acid and amino acid derivatives

Amino acid and amino acid derivatives separation was achieved through the use of a Scherzo SM-C18 column 3 μm, 3.0 × 150 mm (Imtakt Corp, Japan) maintained at 10 °C. The chromatographic gradient started at 100% mobile phase A (0.2% formic acid in water) for 2 min post sample injection. The hold was followed with a 6 min gradient to 80% mobile phase B (0.2% formic acid in methanol) at a flow rate of 0.4 ml/min. This was followed by a 5 min hold time at 100% mobile phase B and a subsequent re-equilibration time (6 min) before next injection. A sample volume of 5 μl of sample was injected for analysis. Sample temperature was maintained at 4 °C before injection. Eluting compounds were detected by ESI in both positive and negative ionization modes and dMRM as optimized on authentic standards. ESI source conditions were the same as for the nucleotides’ analysis.

### Determination of mouse blood glucose levels

Blood was collected from mice aged 4–8 months old via submandibular bleeding using heparinized microhematocrit capillary tubes (Fisher #22-362-566) (*n* = 5 for each group of mice). Blood collected was applied onto the test strip of the Accu-chek Guide Meter. Statistical analysis performed using Student’s *t* test.

### Reporting summary

Further information on experimental design is available in the Nature Research Reporting Summary linked to this paper.

## Supplementary information

Supplementary Information

Peer Review File

Description of Additional Supplementary Information

Supplementary Data 1

Supplementary Data 2

Supplementary Data 3

Supplementary Data 4

Supplementary Data 5

Supplementary Data 6

Supplementary Data 7

Supplementary Data 8

Supplementary Data 9

Supplementary Data 10

Supplementary Data 11

Supplementary Data 12

Supplementary Data 13

Reporting Summary

## Data Availability

All data needed to evaluate the conclusions in this study are present in the paper and/or its Supplementary Materials. ChiP-Sequencing and RNA-Sequencing data supporting the findings of this study have been deposited into the National Center for Biotechnology Information (NCBI) Gene Expression Omnibus under accessions GSE116905 and GSE116906, respectively. Metabolomics data from LC/MS and GC/MS are deposited in the Metabolights database under Study#MTBLS1639. *PRDM15* levels in Human specimens and cell line samples were extracted from the Publicly available datasets GEO/SRA datasets and Broad CCLE-https://portals.broadinstitute.org/ccle.
