# Supplementary material for: PRDM15 is a key regulator of metabolism critical to sustain B-cell lymphomagenesis
Source: Nat Commun. 2020 Jul 14;11:3520. doi: 10.1038/s41467-020-17064-0 (PMC7360777; doi:10.1038/s41467-020-17064-0)
Supplement: Supplementary file 1 — Supplementary Information [file 41467_2020_17064_MOESM1_ESM.pdf]

Supplementary Fig.1

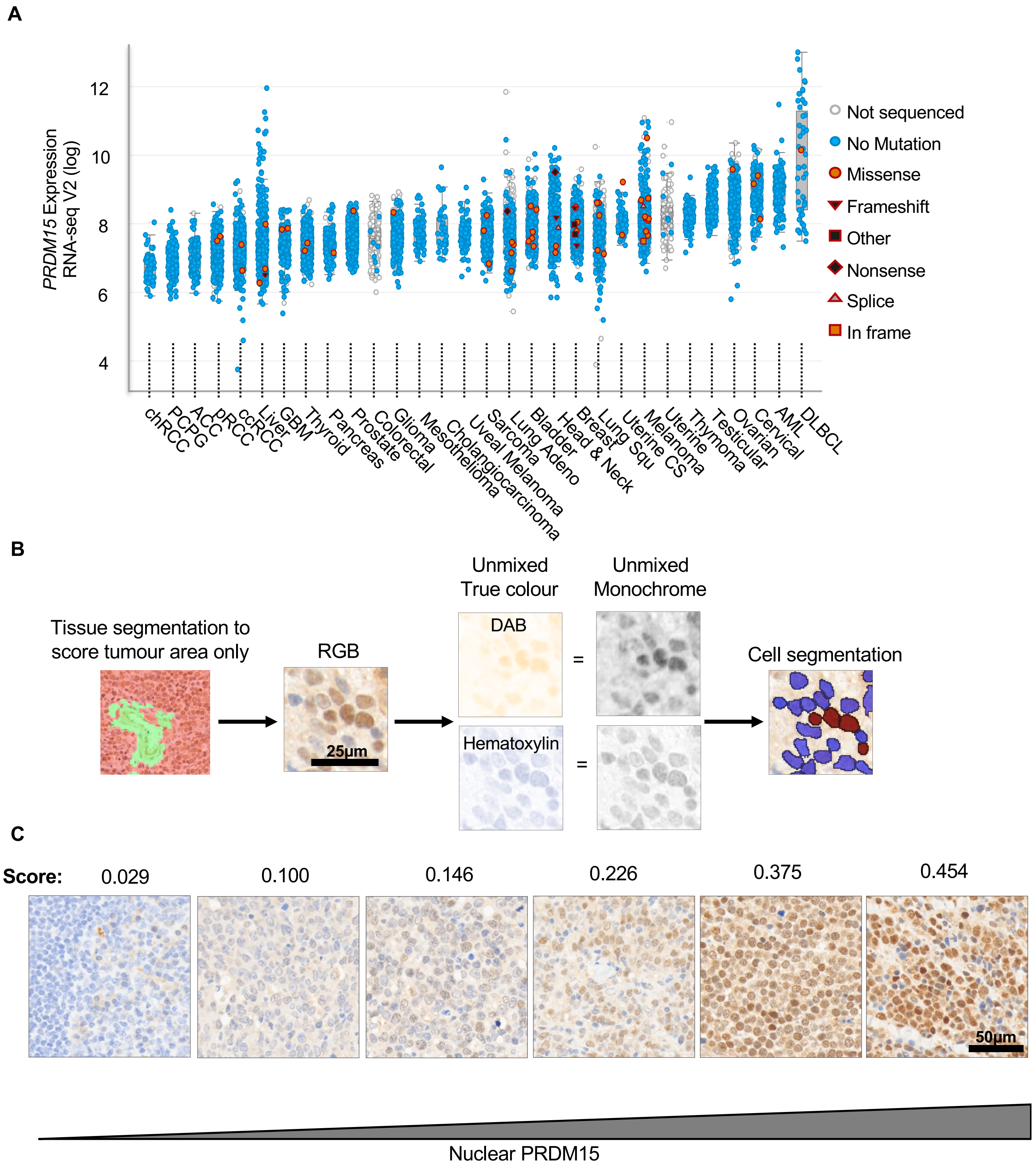

Supplementary Figure 1. A. Expression of human PRDM15 in multiple cancer types (source: TGCA-http://www.cbioportal.org). **B.** Schematic representation of quantitative IHC. Bright field images of tonsil and lymphoma samples acquired by a multispectral microscope were subjected to trainable tissue segmentation to define regions of interest (i.e. malignant cells vs. blood vessels in lymphoma). Multispectral IHC images were unmixed using a spectral library to extract monochrome components of DAB and haematoxylin. Haematoxylin counterstaining was next used to create a nuclear mask of cells within regions of interest, for which a mean nuclear intensity score of DAB was measured. **C.** Example of PRDM15 IHC images with respective mean nuclear DAB optical density (OD, peak weighting) values. Panels B-C describe the methodology used for quantitative IHC experiments (n=1).

Supplementary Fig.2

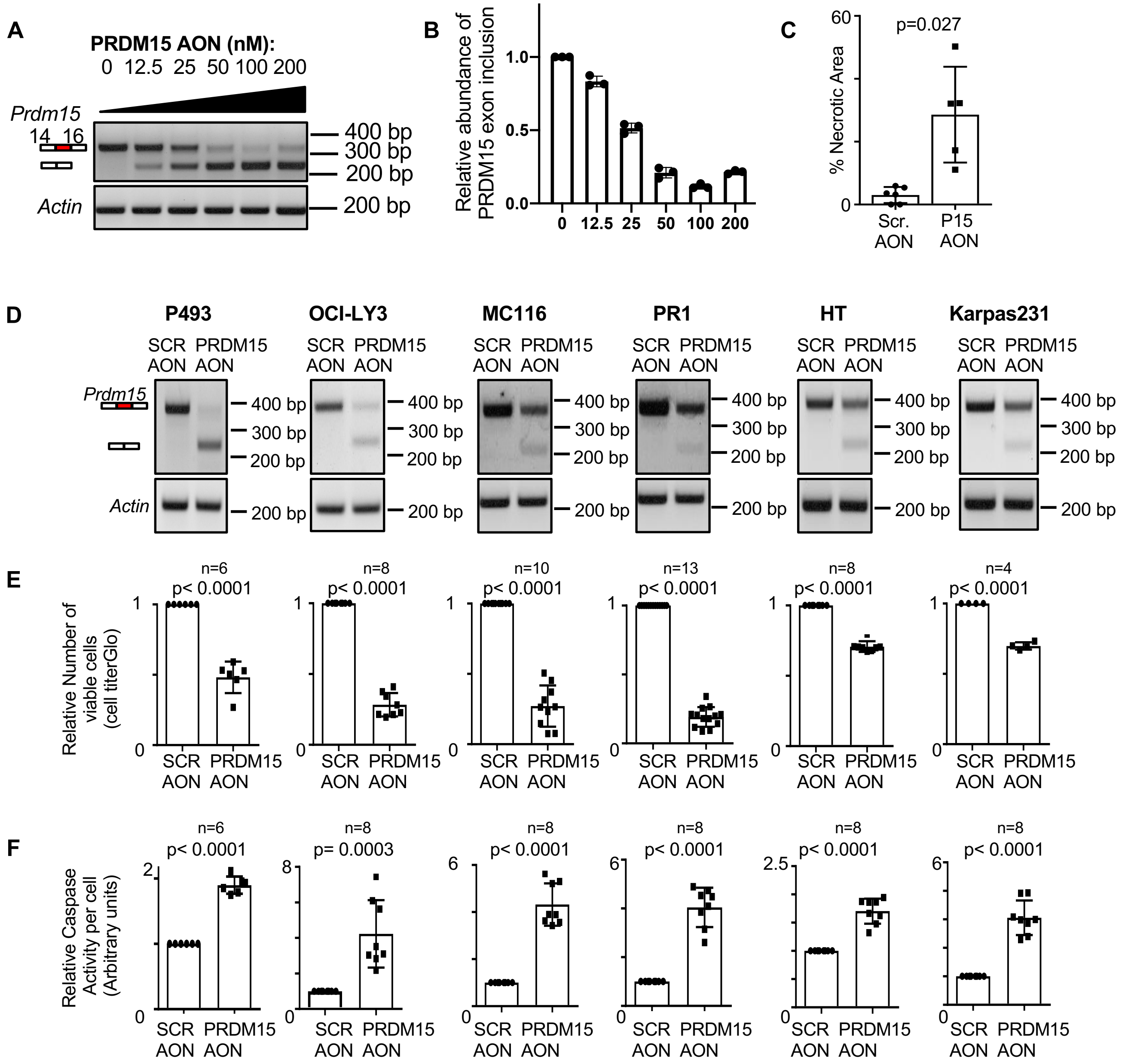

Supplementary Figure 2. A. Human DLBCL cells were transfected with the indicated amount of the PRDM15 AON to calculate the effective IC50 value. RNA was collected and the efficacy of exon skipping was measured by semi-quantitative PCR. The forward and reverse primers were designed on Exons 14 and 16, respectively, so as to amplify a longer isoform (top band) containing exons 14, 15 and 16, and a shorter isoform (generated by exon skipping induced by PRDM15-AON) lacking exon 15. *Actin* is used as a loading control. **B.** Quantification of the experiment in panel A (n=3 technical replicates). **C.** Quantification of the necrotic area in panel 1I. **D-F.** The indicated B cell lymphoma lines (P493, OCI-LY3 (ABC DLBCL), MC116, PR1, HT and Karpas231 (GBC DLBCL) were electroporated with the indicated AONs (either scramble control of targeting PRDM15). **D.** Semi-quantitative PCR and agarose gel electrophoresis. The forward and reverse primers were designed on *PRDM15* Exons 14 and 16, respectively, so as to amplify a longer isoform (normal) containing exons 14, 15 and 16, and a shorter isoform (generated by exon skipping induced by PRDM15 AON) lacking exon 15. **E.** Relative viability and **F.** Relative Caspase 3/7 activity in the indicated B cell lymphoma lines 3 days following electroporation with the indicated AONs. In panels D-E, data are from different biological replicates (the number "n" is indicated in the respective panels). Center values, mean; error bars, s.d. Student's *t* test (two-sided) was used for statistical analysis.

Supplementary Fig.3

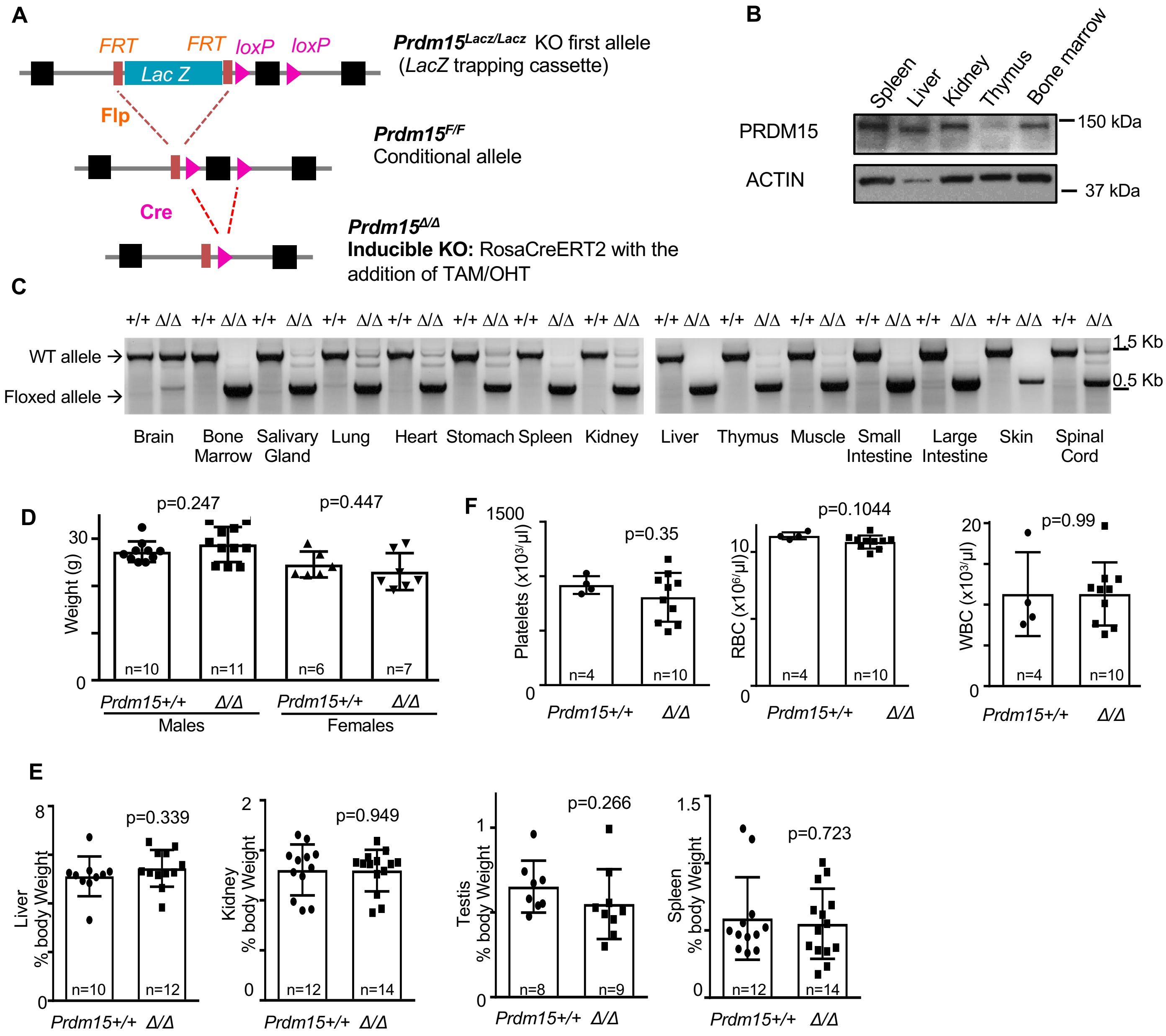

Supplementary Figure 3. A. Schematic representation of the *Prdm15* 'knockout-first' conditional allele (adapted from EUCOMM). **B.** Western blot of PRDM15 protein in the indicated adult mouse tissues (n=2). **C.** Validation of efficacy and durability of tamoxifen-induced Cre-mediated recombination of the PRDM15 locus at 4 months after tamoxifen injection. Primers were designed to amplify a product spanning the floxed locus. The upper band represents the wildtype allele and the lower band represents the floxed allele. **D.** Weights of 4-month-old *Prdm15$^{+/+}$;R26* and *Prdm15$^{F/F}$;R26* mice that were injected with tamoxifen at 8-weeks of age. **E.** Weights of the indicated organs of PRDM15$^{+/+}$ and PRDM15$^{\Delta/\Delta}$ mice 2 months after tamoxifen injection. **F.** Platelets, red and white blood cell counts in PRDM15$^{+/+}$ and PRDM15$^{\Delta/\Delta}$ mice 2 months after tamoxifen injection. In panels D-F, each data point is one mouse (the number "n" is indicated in the respective panels). Center values, mean; error bars, s.d. Student's *t* test (two-sided) was used for statistical analysis.

Supplementary Fig.4

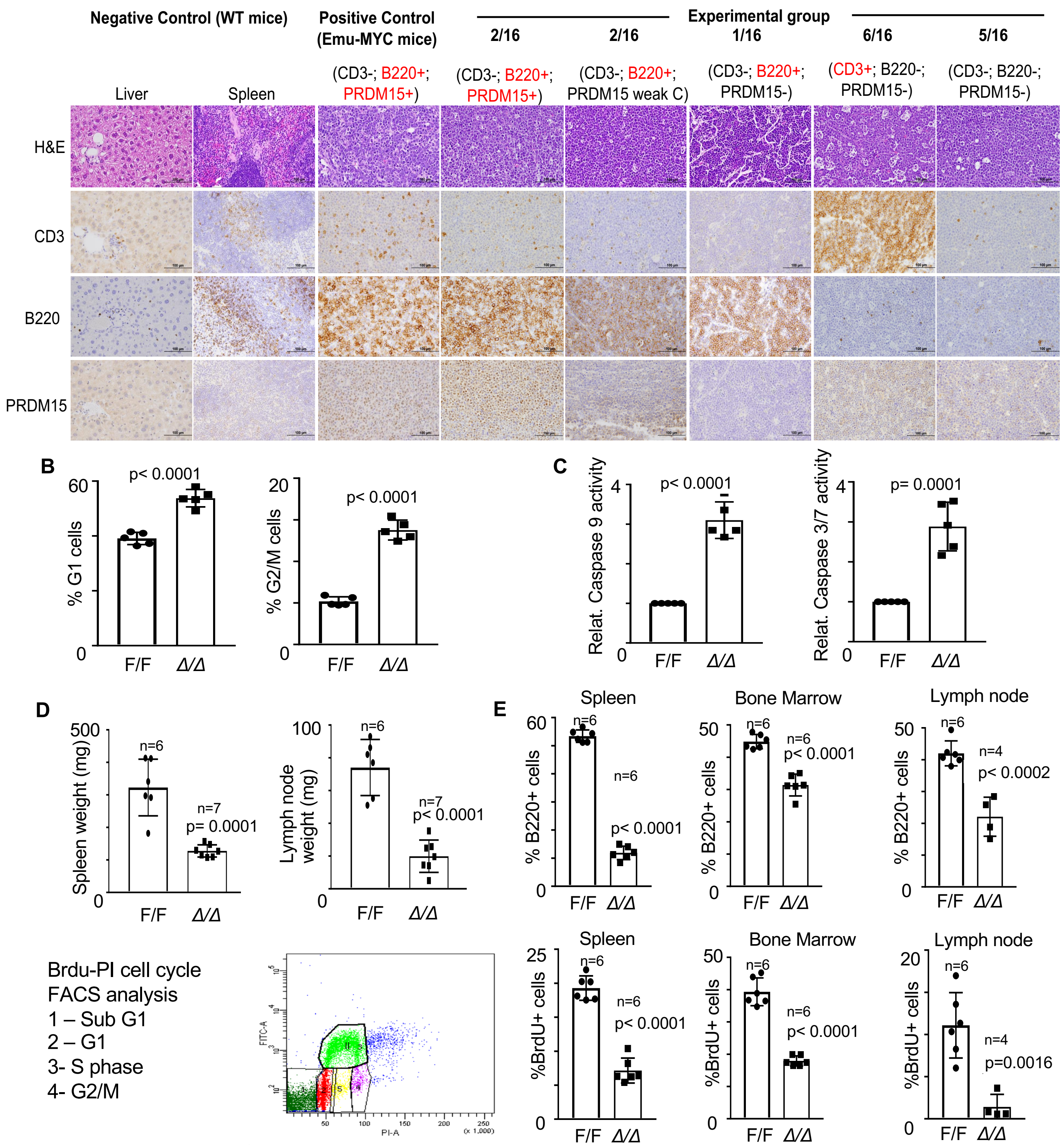

Supplementary Figure 4. A. Representative histology images showing H&E; B220, CD3 and PRDM15 staining of a Wild Type (WT) spleen and tumors recovered from *Prdm15$^{F/F}$;R26; Eμ-Myc mice treated with Corn oil (Prdm15$^{F/F}$) or TAM (Prdm15$^{\Delta/\Delta}$)*. **B-C.** *Prdm15$^{F/F}$;R26; Eμ-Myc* tumor cells were cultured in vitro and 4-OHT (OHT) was added to induce PRDM15 deletion (Δ/Δ). We quantified **B.** % of cells in different phases of the cell cycle and **C.** Relative increase in caspase 9 and 3/7 activity following PRDM15 depletion. In B-C, data are from independent primary lines (n=5). **D.** Spleen and lymph node weight of mice treated with Tamoxifen –relative to Fig.3G. **E.** %B220$^+$ B cells (top panel) and BrdU$^+$ cells (bottom panel) in Bone Marrow (BM), Spleen (SP) and Lymph nodes (LN) of mice treated with Tamoxifen –relative to Fig.3G; the lower left panel displays an example of the gating strategy used. The number "n" in panels D-E indicated the number of mice used to collect the data. In panels B-E, center values, mean; error bars, s.d. Student's *t* test (two-sided) was used for statistical analysis.

Supplementary Fig.5

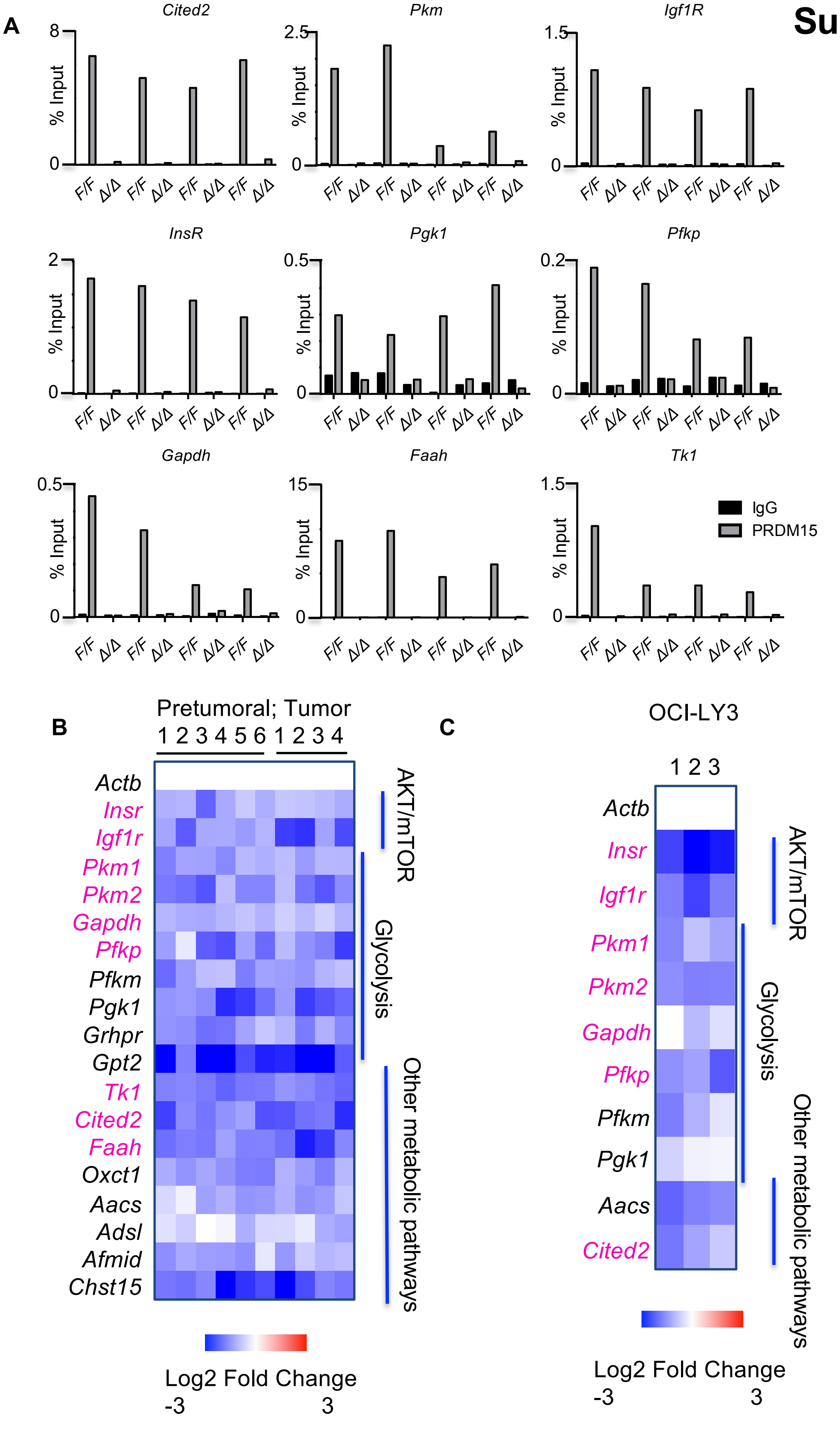

Supplementary Figure 5. A. The enrichment of PRDM15 binding to selected glycolytic targets identified by ChIP-seq were validated in tumor samples from 4 independent mice by Chip qPCR. **B.** The downregulation of selected glycolytic targets identified by RNA-seq were validated in pre-tumoral bone marrow samples (n=6 independent mice) or tumor samples (n=4 independent mice) following PRDM15 depletion *in vitro*. Direct PRDM15-targets are highlighted in red. **C.** The expression of various glycolytic genes in the human DLBCL cell line OCI-LY3 following PRDM15 AON transfection was measured by qPCR. Direct PRDM15-targets are highlighted in red.

Supplementary Fig.6

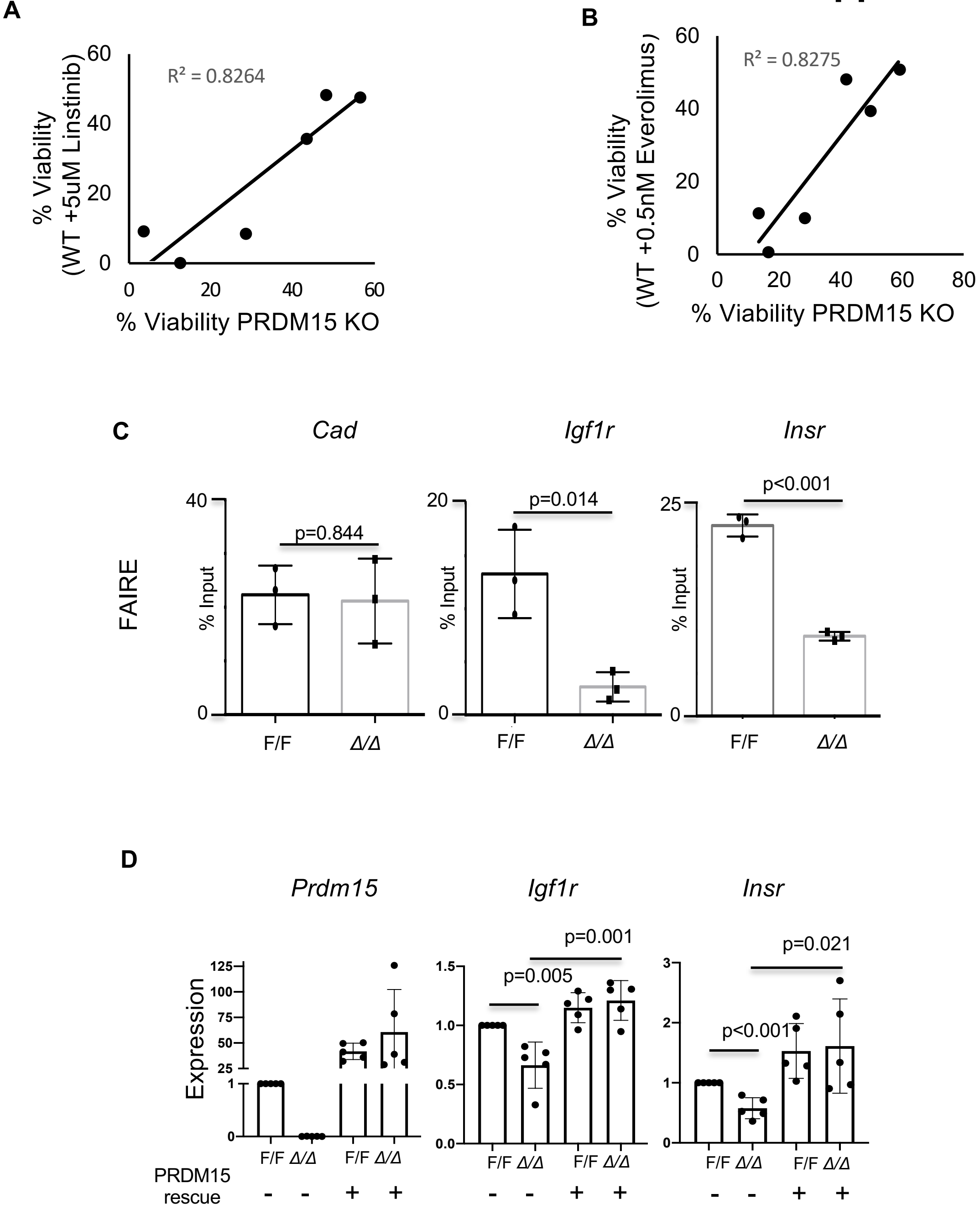

Supplementary Figure 6. A-B. Scatter plots showing a strong correlation between the sensitivity of the *Eµ-Myc* tumor lines to PRDM15 depletion and **(A)** linsitinib (IGF1R/INR dual inhibitor), or **(B)** everolimus (mTORC1 inhibitor). Each point represents correlation data for *Prdm15*$^{F/F}$*(*Etoh+drug*) vs. Prdm15*$^{\Delta/\Delta}$ *(*OHT+DMSO*)* from an independent *Eµ-Myc* tumoral line (n=6 lines; 3 technical replicates per line). **C.** FAIRE-qPCR quantifies the accessibility of the *Igf1R* and *InsR* promoters upon PRDM15 depletion. Data are collected form independent primary lines (n=3). The *Cad* promoter is used as a negative control. **D.** The expression of *Igf1R* and *InsR upon PRDM15* depletion (Δ/Δ) was assessed by qPCR. Prior to the addition of OHT, cells where additionally transfected either with empty vector control (EV) or a vector expressing PRMD15 as indicated, to assess a rescue in gene expression. Each data point is an independent cell culture (n=4). In (C-D), Center values mean; error bars, s.d. Student's *t* test (two-sided) was used for statistical analysis.

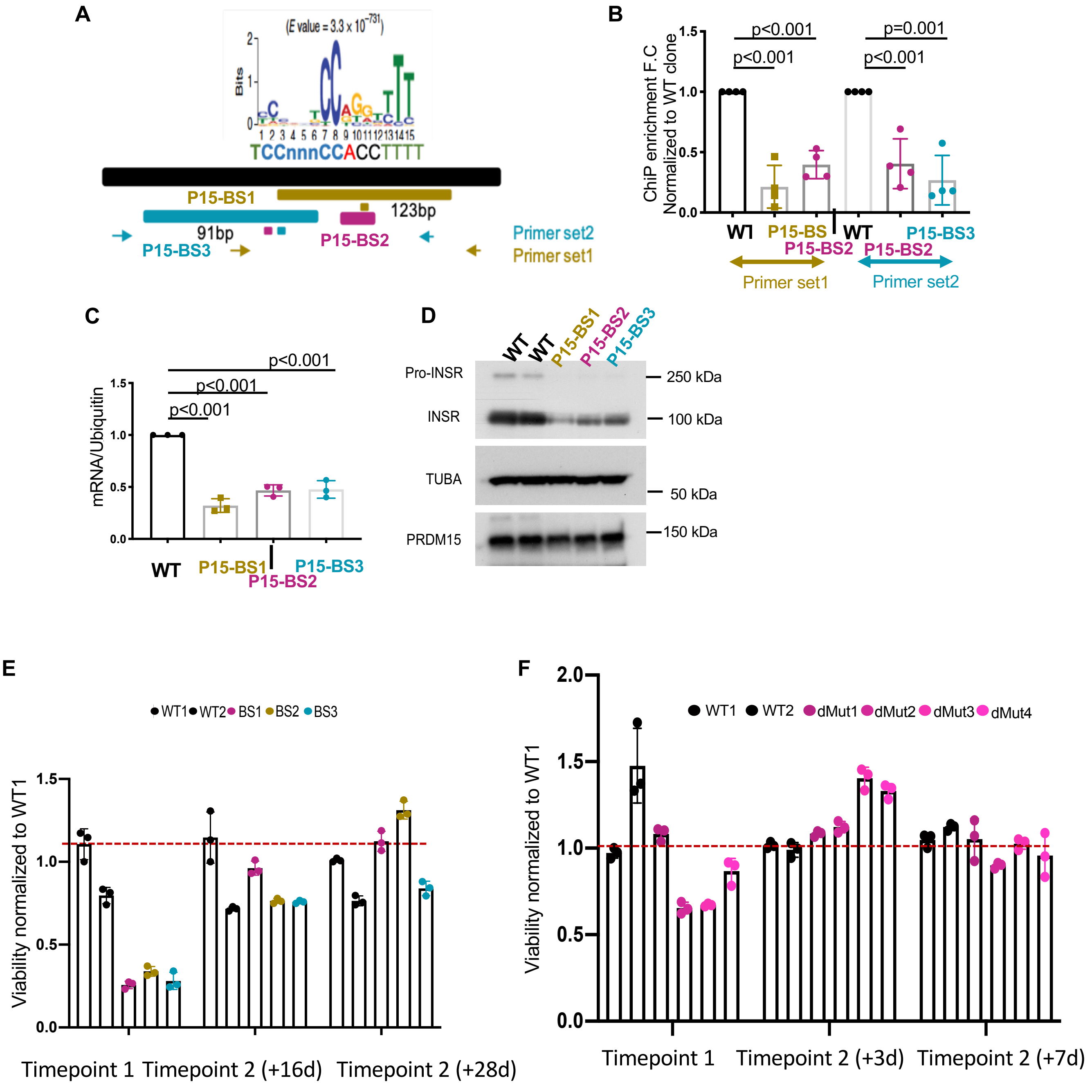

Supplementary Figure 7. A. Schematic representation of the deletions identified in the PRDM15 binding site (BS) within the *Insr* promoter region, in 3 CRSIPR clones. **B**. Assessment of PRDM15 binding to the *Insr* promoter, by ChiP-qPCR, in the CRISPR clones from panel **A**. **C-D.** *Insr* expression levels quantified by qPCR (**C**) and WB (**D**) in the PRDM15-BS mutant clones. **E-F** Cell viability of the *Insr*^{BSKO} (BS) clones **(E)** and the *Insr*^{BSKO}/*Igf1r*^{KO} double mutant (dMut) clones **(F)** assessed by the titer Glow luminescent assay at different timepoints/passages. The *Insr*^{BSKO} were created first and validated, then IGF1R KO subclones were generated from validated *Insr*^{BSKO} clones. In panels B-C, data are from 4 and 3 independent cell cultures, respectively. Center values mean; error bars, s.d. Student's *t* test (two-sided) was used to determine significance. In panel E-F, data are from 3 technical replicates; the independent repeats are shown individually at different timepoints. Center values mean; error bars, s.d.

Supplementary Fig.8

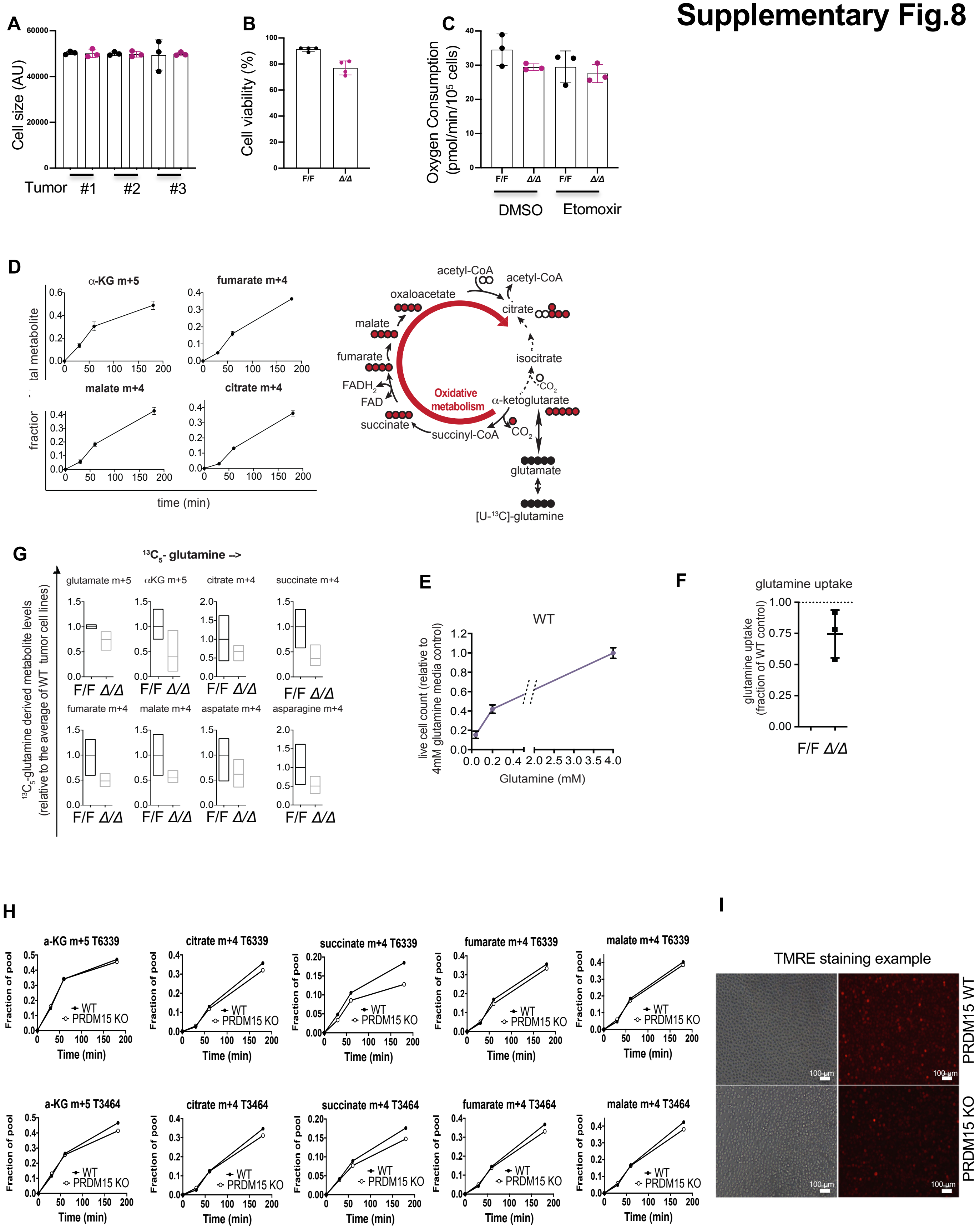

Supplementary Fig. 8. A. The cell size of WT and PRDM15-KO cells from 3 independent tumoral lines was measured by flow cytometry; FSC data are shown (AU – arbitrary units; n=3 technical replicates). Error bars represent SD. **B.** The cell viability of WT and PRDM15-KO cells from an independent tumor line. Error bars represent SD (n=4). **C.** Seahorse β-oxidation assay on WT and PRDM15 KO cells from an independent tumor line. Error bars represent SD (n=3). **D**. WT cells were incubated in media with $^{13}C_5$-glutamine for 30, 60 and 180min. Stable isotope tracer analysis was performed by GC/MS, and the fraction of the indicated is shown relative to the total pool of the respective metabolite (left panel). Error bars represent SD (n=3 tumoral lines). Right panel: schematic representation of the ^{13}C atom incorporation into metabolites. **E**. After PRDM15 KO induction, cells were seeded in glutamine-free media supplemented with glutamine for 48h. Live cells were counted, and data are expressed as a fraction of 4mM glutamine (full) media. Error bars represent SD (n=3 tumoral lines). **F**. One day post PRDM15 KO-induction, *Prdm15*$^{F/F}$ and *Prdm15*$^{\Delta/\Delta}$ cells were seeded in fresh media. 24h later, glutamine concentration in the cellular media were measured and subtracted from that of media alone. Glutamine uptake is shown relative to the WT cells. Error bars represent SD (n=3 tumoral lines). **G**. One day post PRDM15 KO-induction, WT and *Prdm15*KO cells were incubated in media with $^{13}C_5$-glutamine for 180 min. Stable isotope tracer analyses were performed and the levels of indicated isotopomers ion amounts are shown as absolute amount and relative to the average of WT cells (n=3 tumors). **H**. Data of the fraction of ^{13}C labeled metabolites relative to the total pool is shown. One day post KO-induction, cells are incubated with $^{13}C_5$-glutamine. Stable isotope tracer analysis was performed by GC/MS, and the fraction of the indicated isotopomers is shown relative to the total pool of the respective metabolite (left panel); data from 2 independent tumoral lines is shown. **I.** Representative microscopy image for the TMRE staining relative to Fig. 6E (Scale bar:100μm) (n=6).
