# Supplementary material for: PRDM15 is a key regulator of metabolism critical to sustain B-cell lymphomagenesis
Source: Nat Commun. 2020 Jul 14;11:3520. doi: 10.1038/s41467-020-17064-0 (PMC7360777; doi:10.1038/s41467-020-17064-0)
Supplement: Supplementary file 3 — Description of Additional Supplementary Information [file 41467_2020_17064_MOESM3_ESM.pdf]

Description of Additional Supplementary Files

File Name: Supplementary Data 1
Description: IHC staining of Eμ-Myc tumors.

File Name: Supplementary Data 2
Description: Prdm15-bound sites in tumor samples.

File Name: Supplementary Data 3
Description: Differential expression in tumor samples.

File Name: Supplementary Data 4
Description: Genes that were up-regulated in at least 2 tumors.

File Name: Supplementary Data 5
Description: Gene Ontology and KEGG pathway analysis of genes that were up-regulated in at least 2 tumors.

File Name: Supplementary Data 6
Description: Genes that were down-regulated in at least 2 tumors.

File Name: Supplementary Data 7
Description: Gene Ontology and KEGG pathway analysis of genes that were down- regulated in at least 2 tumors.

File Name: Supplementary Data 8
Description: Genes that were bound and regulated in at least 2 tumors.

File Name: Supplementary Data 9
Description: Gene Ontology and KEGG pathway analysis of genes that were bound and regulated in at least 2 tumors.

File Name: Supplementary Data 10
Description: Primer sequences.

File Name: Supplementary Data 11
Description: Sequence of the sgRNAs used for CRISPR/Cas9 editin.

File Name: Supplementary Data 12
Description: Antibody information.

File Name: Supplementary Data 13
Description: Uncropped gels.
