# Supplementary figures and images for: PRDM15 is a key regulator of metabolism critical to sustain B-cell lymphomagenesis
Source: Nat Commun. 2020 Jul 14;11:3520. doi: 10.1038/s41467-020-17064-0 (PMC7360777; doi:10.1038/s41467-020-17064-0)

Fig. 5a

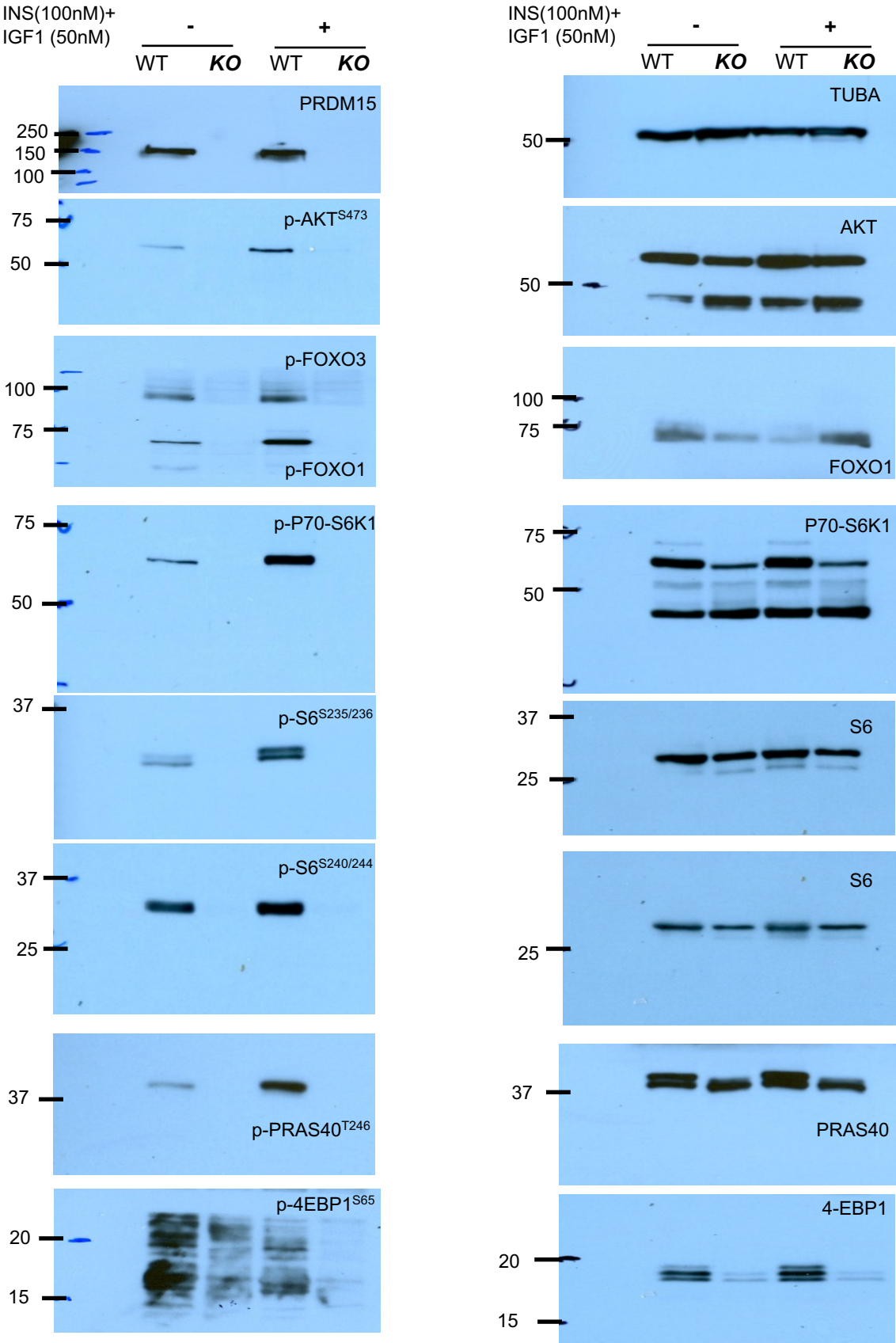

Fig. 5b

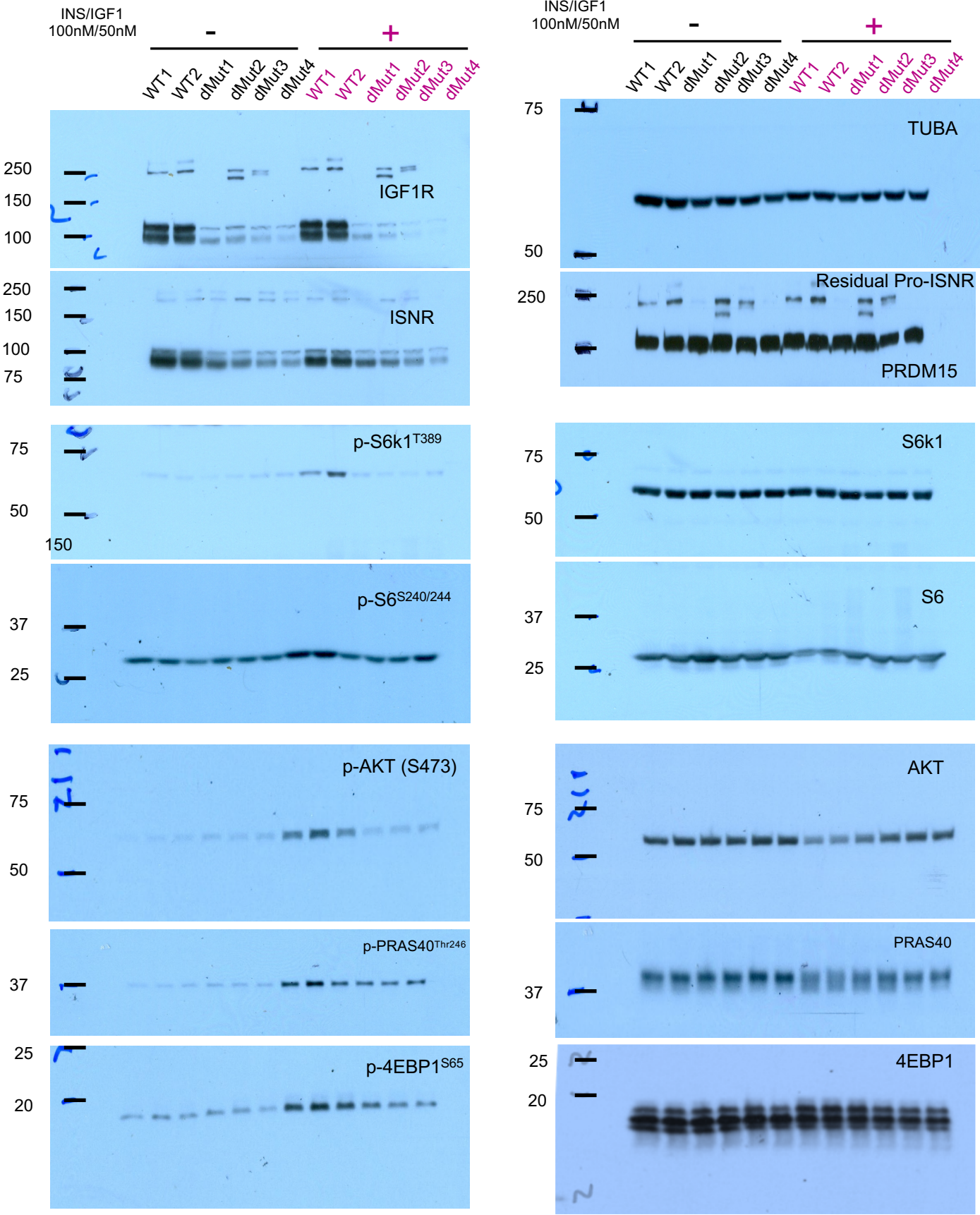

**Fig. 5c**

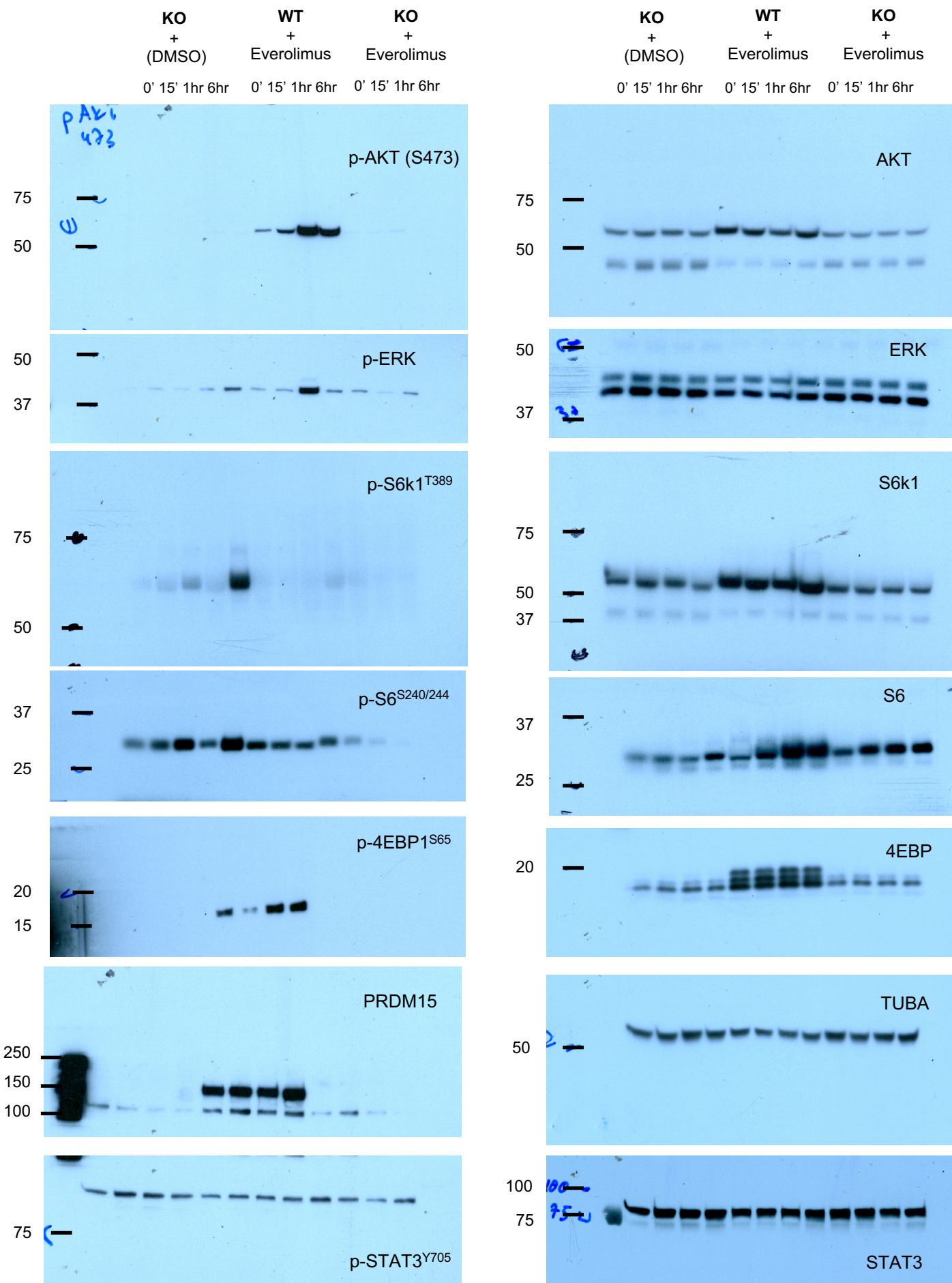

Supp. Fig. 7d

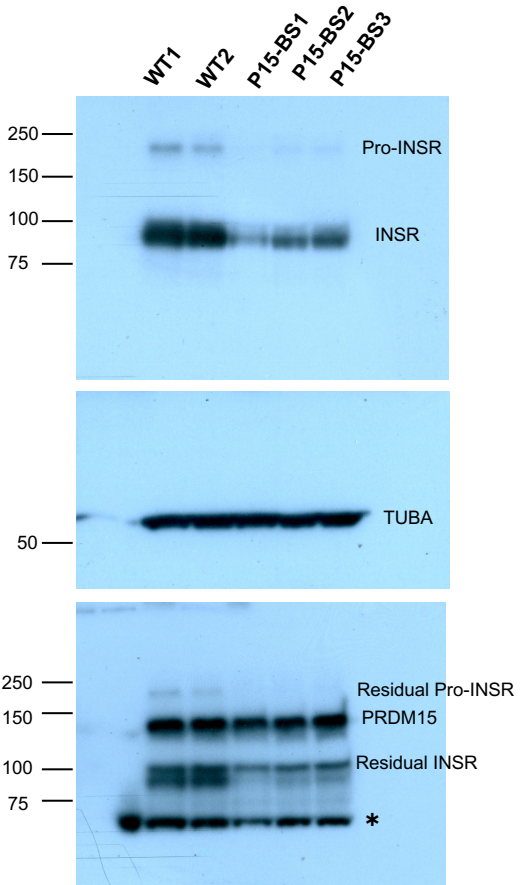

Fig. 3a

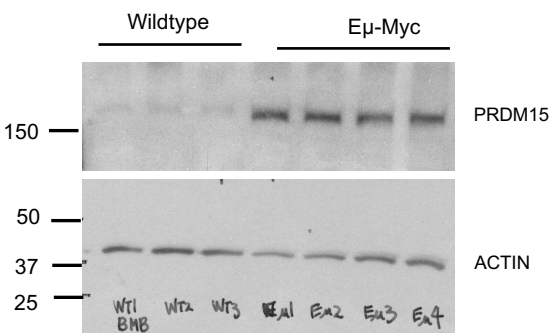

Supp. Fig 3b

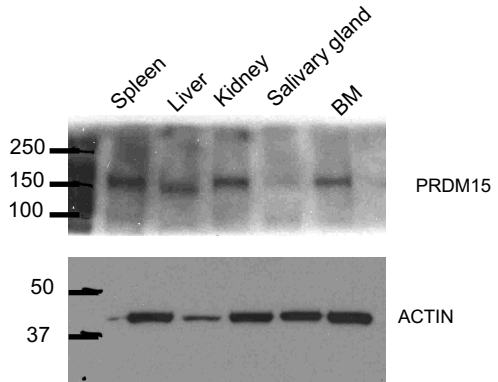

Fig. 1e

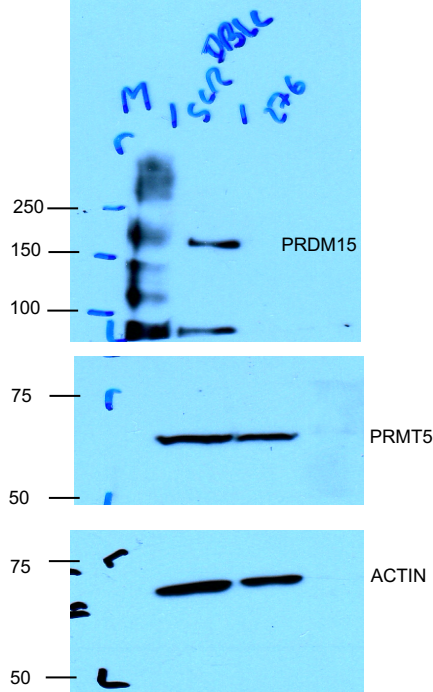

Fig. 3c

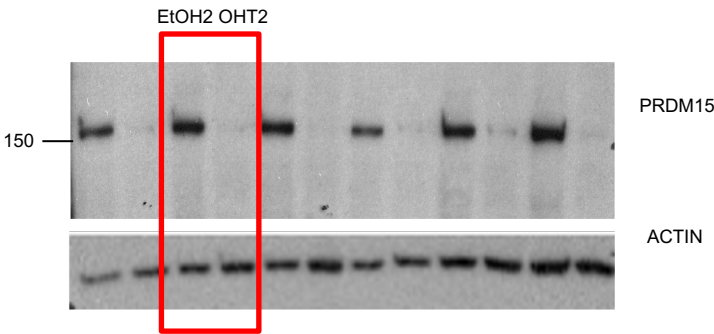

\* unspecific band

Supp. Fig. 2a

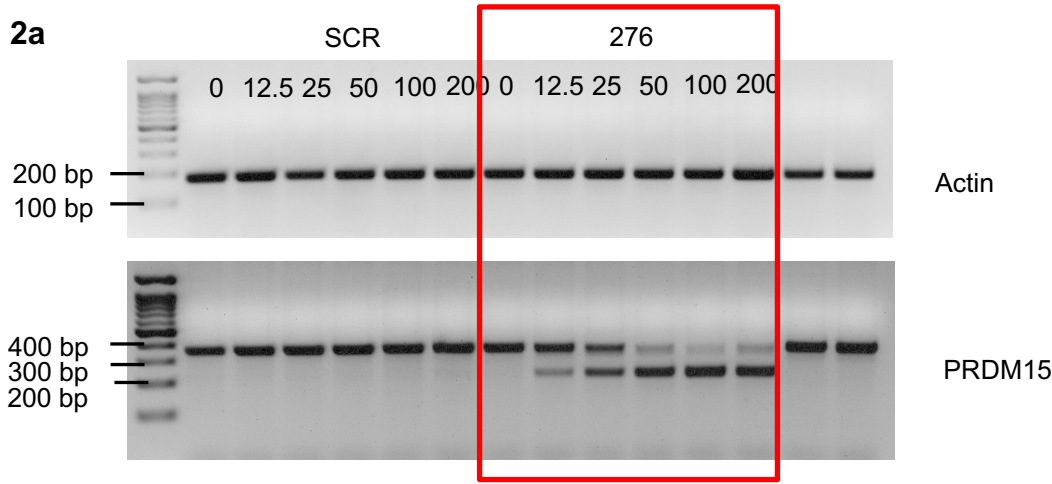

Supp. Fig. 2c

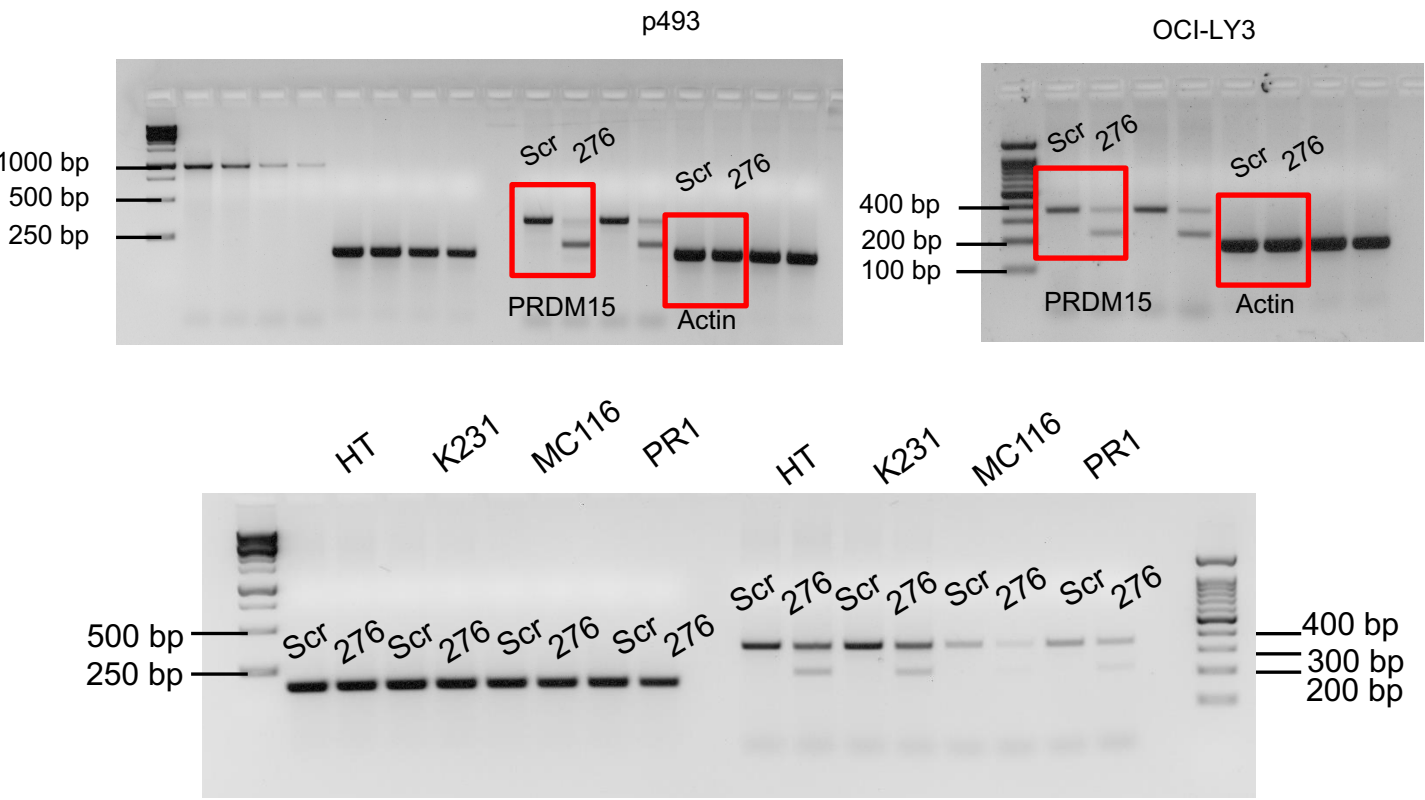

Supp. Fig. 3c

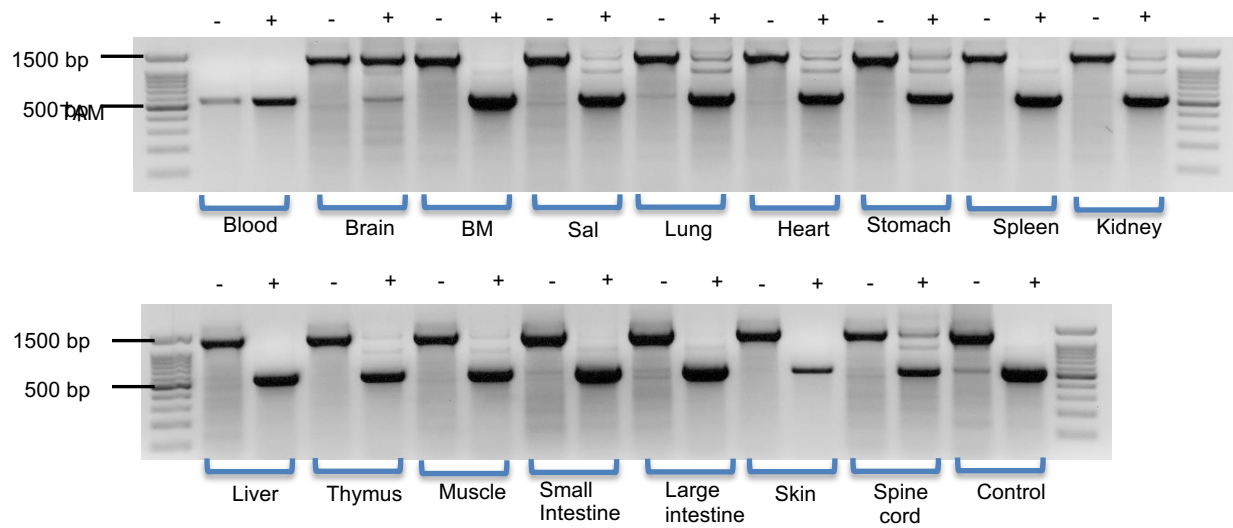

Supplement: Supplementary file 16 — Supplementary Data 13 [file 41467_2020_17064_MOESM16_ESM.pdf]
