# Supplementary material for: PRDM15 is a key regulator of metabolism critical to sustain B-cell lymphomagenesis
Source: Nat Commun. 2020 Jul 14;11:3520. doi: 10.1038/s41467-020-17064-0 (PMC7360777; doi:10.1038/s41467-020-17064-0)
Supplement: Supplementary file 17 — Reporting Summary [file 41467_2020_17064_MOESM17_ESM.pdf]

nature research

Corresponding author(s): *Ernesto Guccione*

Reporting Summary

Nature Research wishes to improve the reproducibility of the work that we publish. This form provides structure for consistency and transparency in reporting. For further information on Nature Research policies, see Authors & Referees and the Editorial Policy Checklist.

Statistical parameters

When statistical analyses are reported, confirm that the following items are present in the relevant location (e.g. figure legend, table legend, main text, or Methods section).

n/a	Confirmed	
☐	☒	The exact sample size (*n*) for each experimental group/condition, given as a discrete number and unit of measurement
☐	☒	An indication of whether measurements were taken from distinct samples or whether the same sample was measured repeatedly
☐	☒	The statistical test(s) used AND whether they are one- or two-sided<br>*Only common tests should be described solely by name; describe more complex techniques in the Methods section.*
☐	☒	A description of all covariates tested
☐	☒	A description of any assumptions or corrections, such as tests of normality and adjustment for multiple comparisons
☐	☒	A full description of the statistics including central tendency (e.g. means) or other basic estimates (e.g. regression coefficient) AND variation (e.g. standard deviation) or associated estimates of uncertainty (e.g. confidence intervals)
☐	☒	For null hypothesis testing, the test statistic (e.g. *F*, *t*, *r*) with confidence intervals, effect sizes, degrees of freedom and *P* value noted<br>*Give P values as exact values whenever suitable.*
☒	☐	For Bayesian analysis, information on the choice of priors and Markov chain Monte Carlo settings
☒	☐	For hierarchical and complex designs, identification of the appropriate level for tests and full reporting of outcomes
☒	☐	Estimates of effect sizes (e.g. Cohen's *d*, Pearson's *r*), indicating how they were calculated
☐	☒	Clearly defined error bars<br>*State explicitly what error bars represent (e.g. SD, SE, CI)*

Our web collection on statistics for biologists may be useful.

Software and code

Policy information about availability of computer code

Data collection	n.a.
Data analysis	inForm 2.2 software, Primer3 software 040, Bowtie 0.12.8, STAR v2.4.2a, EdgeR version 3.28.1 released under Bioconductor 3.10.

For manuscripts utilizing custom algorithms or software that are central to the research but not yet described in published literature, software must be made available to editors/reviewers upon request. We strongly encourage code deposition in a community repository (e.g. GitHub). See the Nature Research guidelines for submitting code & software for further information.

Data

Policy information about availability of data

All manuscripts must include a data availability statement. This statement should provide the following information, where applicable:
- Accession codes, unique identifiers, or web links for publicly available datasets
- A list of figures that have associated raw data
- A description of any restrictions on data availability

The raw data is available as GSE116905 (ChIP-seq), GSE116906 (RNA-seq) and Study#MTBLS1639 (metabolomics).

Field-specific reporting

Please select the best fit for your research. If you are not sure, read the appropriate sections before making your selection.

☒ Life sciences ☐ Behavioural & social sciences ☐ Ecological, evolutionary & environmental sciences

For a reference copy of the document with all sections, see nature.com/authors/policies/ReportingSummary-flat.pdf

Life sciences study design

All studies must disclose on these points even when the disclosure is negative.

Sample size	No sample size calculation was performed.
Data exclusions	No data was excluded from the analyses.
Replication	All experiments were repeated at least thrice. For studies involving primary cells from mice, cells were derived from multiple independent mice.
Randomization	For mouse studies, animals were gender and age matched.
Blinding	No blinding was carried out.

Reporting for specific materials, systems and methods

Materials & experimental systems

n/a	Involved in the study
☐	☒ Unique biological materials
☐	☒ Antibodies
☐	☒ Eukaryotic cell lines
☒	☐ Palaeontology
☐	☒ Animals and other organisms
☒	☐ Human research participants

Methods

n/a	Involved in the study
☐	☒ ChIP-seq
☐	☒ Flow cytometry
☒	☐ MRI-based neuroimaging

Unique biological materials

Policy information about availability of materials

Obtaining unique materials	n.a.

Antibodies

Antibodies used

α

PRDM15 In house (doi: 10.1038/ng.3922)
-TUBULIN T5168 (Sigma)
Phosph -Akt (Ser47) CST#4060
Akt CST#9272
phoshph Foxo1(thr24)/Foxo3a(thr32) CST#9464
Phospho-FoxO1 (Ser256) CST#9461
FoxO1 CST#2880
PRAS40 CST2691
Phospho-PRAS40 CST#2997
IGF1RZI001(ThermoFisher#39-6700)

p-STAT3	CST#9145
pP70 S6 Kinase (Thr389)	CST#9234
pS6 (Ser235/236)	CST#2211
pS6 (S240-44)	CST#5364
p-4E-BP1 (Ser65)	CST#9451
STAT3	CST#9139
INSR	CST#3025
P70 S6 Kinase	CST#2708
S6	CST#2217
4E-BP1 (53H11)	CST#9644
PARP	CST#9542
c-Casp3	CST#9661

Validation

anti-PRDM15: validation described in Mzoughi et al Nat. Genetics 2017. all the others were tested in insulin stimulated or starved cells for their specificity

Eukaryotic cell lines

Policy information about cell lines

Cell line source(s)	OCI-LY3, MC116, Karpas 231, HT and PR1 cells were a kind gift from Dr. Ong Sin Tiong (Duke NUS) and originally purchased from ATCC.
Authentication	No cell line authentication was performed.
Mycoplasma contamination	All cell lines tested negative
Commonly misidentified lines (See ICLAC register)	No commonly misidentified lines were used

Animals and other organisms

Policy information about studies involving animals; ARRIVE guidelines recommended for reporting animal research

Laboratory animals	Mouse- C57/Bl6 and SCID. Both male and female mice were used as recipients, typically between 4-6 weeks old.
Wild animals	This study did not involve wild animals.
Field-collected samples	This study did not involve field-collected samples.

ChIP-seq

Data deposition

☒ Confirm that both raw and final processed data have been deposited in a public database such as GEO.

☐ Confirm that you have deposited or provided access to graph files (e.g. BED files) for the called peaks.

Data access links *May remain private before publication.*	GSE116905
Files in database submission	GSM4466725_Eu_MYC_input_mm9<br>GSM4466726_Eu_MYC_IP_mm9<br>_
Genome browser session (e.g. UCSC)	can be uploaded from GSE116905. GSE116905_RAW.tar

Methodology

Replicates	3 (extensive validation on multiple biological replicates by qPCR)
Sequencing depth	>60mln
Antibodies	PRDM15 In house (doi: 10.1038/ng.3922)
Peak calling parameters	For ChIP-sequencing, DNA libraries were prepared using the TruSeq ChIP Sample Prep Kit (IP-202-1012), following the manufacturer's instructions, and sequenced in the Illumina Hiseq 2000 and Nextseq 500 at the Genome Institute Singapore (GIS). The sequenced reads were mapped to mm9 build of the mouse genome from University of California Santa Cruz (UCSC) genome database using Bowtie 0.12.8 26 with default parameters except –m 1 and –segment-mismatches 2. Only reads which mapped uniquely to the genome with at most two mismatches were kept. Duplicate reads were filtered by MACS (2.10) 27 to limit PCR-induced biases and the q-value was set to 0.05 for peak calling.
Data quality	Annotations of nearby genes associated with PRDM15 peaks were performed using GREAT (2.02) 28. Each gene was assigned a basal regulatory domain defined as prompter region (-5kb/+5kb), and a distal regulatory region (50kb) extended in both directions. K-means clustering of PRDM15 peaks and histone modifications in mESCs were performed with seqMINER 29.<br>Gene ontology analysis of associated genes was performed using Metacore 30 The associated GO terms were ranked by p-value. 200bp wide DNA sequences of PRDM15 peak regions were used to identify enriched motifs. The enriched motifs were discovered by using the Multiple Em For Motif Elicitations (MEME) software suite (http://meme.nbcr.net/memecgi-bin/meme-chip.cgi) 31 with default settings. Reported motifs were ranked according to E-value significance, which is the estimated probability of the expected number of motifs with the given log-likelihood ratio compared to a random set of sequences of similar size and sequence width. Series records GSE116905 and GSE116906 provide access to all data presented in this manuscript.

Software	Only reads which mapped uniquely to the genome with at most two mismatches were kept. Duplicate reads were filtered by MACS (2.10) 27 to limit PCR-induced biases and the q-value was set to 0.05 for peak calling.

Flow Cytometry

Plots

Confirm that:

☒ The axis labels state the marker and fluorochrome used (e.g. CD4-FITC).

☒ The axis scales are clearly visible. Include numbers along axes only for bottom left plot of group (a 'group' is an analysis of identical markers).

☒ All plots are contour plots with outliers or pseudocolor plots.

☒ A numerical value for number of cells or percentage (with statistics) is provided.

Methodology

Sample preparation	This is detailed in the Materials and Methods section.
Instrument	BD LSR II
Software	BD FACS DIVA
Cell population abundance	Cell sorting was not carried out.
Gating strategy	These are shown in representative examples (attached)

☒ Tick this box to confirm that a figure exemplifying the gating strategy is provided in the Supplementary Information.
