# Supplementary material for: PRDM15 is a key regulator of metabolism critical to sustain B-cell lymphomagenesis
Source: Nat Commun. 2020 Jul 14;11:3520. doi: 10.1038/s41467-020-17064-0 (PMC7360777; doi:10.1038/s41467-020-17064-0)
Supplement: Supplementary file 2 — Peer Review File [file 41467_2020_17064_MOESM2_ESM.pdf]

Editorial Note: This manuscript has only been reviewed at another journal that is not operating a transparent peer review scheme.
